# Disrupted neural response timing and duration during hand movement preparation in schizophrenia spectrum disorder: An fMRI study

**DOI:** 10.1038/s41598-026-50969-2

**Published:** 2026-05-02

**Authors:** Harun A. Rashid, Tilo Kircher, Benjamin Straube

**Affiliations:** 1 Department of Psychiatry and Psychotherapy, Marburg University, Rudolf-Bultmann-Strasse 8, 35039 Marburg, Germany; 2 Center for Mind, Brain and Behavior (CMBB), 35039 Marburg, Germany

**Keywords:** Neurology, Neuroscience

## Abstract

Schizophrenia spectrum disorders (SSD) feature deficits in movement intention, predictive mechanisms, and agency. While prior work characterised BOLD amplitude during movement preparation and temporal dynamics across the entire movement period, it remains unknown whether preparatory neural (BOLD) response timing and duration are selectively impaired in SSD. We analysed fMRI data from 20 SSD patients and 20 healthy controls during active and passive hand movements with video feedback (own vs. other hand). Preparatory and executory BOLD responses were modelled separately using the canonical HRF and its temporal (TD) and dispersion (DD) derivatives to evaluate timing and duration. Patients with SSD showed pronounced preparatory abnormalities under own-hand feedback, including delayed responses in right supplementary motor area during active movement preparation. Passive- and active-specific timing was reversed (earlier/later) in the left (caudate, middle temporal gyrus, superior parietal gyrus) and right (postcentral gyrus, right precentral gyrus, anterior cingulate cortex). Response duration showed reduced/reversed modulation (shorter/longer) in the right supplementary motor area, bilateral supramarginal gyrus, left inferior parietal cortex, and bilateral middle temporal gyrus. Earlier response timing in right precentral gyrus during active own-hand were negatively correlated with delusions of being controlled and formal thought disorder. No group differences emerged during execution.

**Supplementary Information:**

The online version contains supplementary material available at 10.1038/s41598-026-50969-2.

## Introduction

Schizophrenia spectrum disorders (SSD) are characterised by disturbances in thought, motor control, and the sense of agency, including difficulties distinguishing self-generated from externally driven actions.^1–7^. Such deficits in sense of agency and ego-demarcation are more pronounced and represent an ideal model to examine in patients with SSD; however, they are also reported in patients with neurological disorders (e.g., stroke, functional neurological disorders).^8–10^ The root of these difficulties likely lies in the disrupted integration of movement intention, motor-sensory outcome predictive mechanisms, distinction between internal and external sensory inputs, and monitoring of actual sensory-motor feedback.^11–16^.

Neurophysiological studies using electroencephalography (EEG) and magnetoencephalography (MEG) studies frequently report altered time-sensitive neural response synchrony in patients with SSD, reflected in impaired oscillatory brain activity in theta, beta, and gamma bands.^2,17–19^ Although spatially limited to cortical regions, EEG/MEG abnormalities have also been reported both before movement onset (readiness potential [RP] and lateralised readiness potential [LRP]) and during or post movement period (event related de/synchronisation [ERD/ERS]), showing alterations in amplitude (reduced or abolished), timing (early/late), or duration.^17,20–22^ EEG/MEG studies have further reported increased temporal imprecision—reduced neural response frequency and phase coherence to stimuli, or decreased phase consistency in spontaneous neural activity.^23^ Such temporal abnormalities—whether delayed, advanced, or desynchronised neural responses across cortico-ponto-thalamo-cerebellar network areas may disrupt the transformation of movement intention (volitional control of agency) into motor commands with sensory feedback predictions, thereby impairing the sense of agency (e.g., passivity experiences) over time.^24–27^ Supporting this notion, fMRI studies in patients with SSD have shown deficits in these network pathways, including aberrant connectivity between the thalamus, pons, and cerebellum.^26,28–31^ However, to date, fMRI studies have not examined whether disruptions involve alterations in the timing and duration of neural responses that primarily occur during movement preparation, execution, or both in patients with SSD.

Typically, analyses of BOLD responses have been conducted using canonical hemodynamic response function (canonical-HRF), which captures amplitudes (more or less engaged neural responses) in subcortical and cortical regions.^15,32^ However, addition of temporal derivative (TD) with canonical-HRF can capture sub-second shift in onset timing relative to canonical HRF, and combination of canonical-HRF + TD + dispersion derivative (DD) can provide shift in duration of neural responses.^33,34^ In this regard, a few studies implemented both TD and DD in HC studies, reporting earlier activation in many brain areas during active versus passive (driven by passive movement device [PMD] itself) movement.^33–35^ Our previous study investigated timing (TD) and duration (DD) abnormalities across the entire hand movement (active versus passive) phase, patients with SSD have shown delayed neural responses in the bilateral insula, left (putamen, thalamus, superior temporal gyrus, postcentral gyrus) and lobule VIII of right cerebellum.^24^ In contrast to the study showing BOLD amplitude abnormalities during the entire hand movement phase,^15^ we demonstrated that patients with SSD are impaired primarily during preparation, not execution by separating these phases.^32^ Furthermore, we have found that there was no significant differences regarding duration of preparation, neither between movement type within groups, nor between groups.^32^ These methodical extensions and novel findings have driven us questioning, if group differences in timing of neural processing between HC and SSD can be explained by distinct timing and duration of BOLD responses during movement preparation or movement execution.^24,34^ However, no prior fMRI study has characterised how temporal dynamics abnormalities across cortical, subcortical, and cerebellar regions are during preparation and execution in patients with SSD. Furthermore, it remains unknown whether specific brain regions have differential timing dynamics that might play a distinct role during the preparatory versus entire movement phase. Clarifying these gaps are so critical to visualise their impaired functional roles across movement types and phases in SSD, especially with a uniform fMRI dataset across studies to build a coherent neuro-mechanistic insight.

To address these unique gaps, this study utilised the uniform fMRI dataset from our previous study employing the same experimental paradigm^24,32^, and isolated preparatory and executory BOLD responses, which were modelled using TD and DD alongside the canonical-HRF analyses (see section: Movement-phase-specific temporal dynamics of BOLD responses). Specifically, we examined questions, compared to HC: (1) Do patients show altered timing (TD) and/or duration (DD) of BOLD responses during hand movement preparation? (2) Which brain regions display earlier/later, shorter/prolonged BOLD responses depending on movement type (active vs. passive) and feedback (own vs. other hand)? and (3) Are these temporal abnormalities associated with core symptoms such as delusions of being controlled (disrupted sense of agency)? Furthermore, we compared findings from this study to our previous study on the entire hand movement phase,^24^ focusing on how timing (TD effects) are characterised during preparatory period, which is distinct from the entire movement phase in patients with SSD.

Unlike our previous studies,^24,32^ the novelty of this study is at least four-fold: first, isolation of movement phases (preparation and execution) and analyses of movement phase specific temporal dynamics (TD and DD) across the brain; second, comparison with TD and DD findings from the entire movement phase^24^; third, relating current TD and DD findings with previous BOLD amplitudes from preparatory and executory phases^32^; and finally, linking movement phase specific abnormalities in timing and amplitude of neural responses with symptoms. Only through segmentation of movement phases (preparation, execution) and analysing them with TD and DD that we did in this study, we can complete the characterisation of movement phase specific abnormalities across the brain, providing new insight into three-dimensional characteristics of neural mechanisms (amplitude, timing, and duration) in patients with SSD.

## Materials and methods

This has been partly explained in our previous study^15,24,32^, here we describe it shortly but in a comprehensive way that is more applicable to this study.

### Clinical and demographic characteristics of included subject’s

Participants were drawn from our previous study^15^ based on strict inclusion/exclusion criteria, and only those participants with high-quality fMRI data (i.e., completed the full fMRI experiment without excessive head motion) were included. This study analysed fMRI data selected from 40 right-handed individuals: 20 patients diagnosed with schizophrenia spectrum disorders (SSD; aged 22 to 56 years; 19 with schizophrenia and one with schizoaffective disorder) and 20 healthy controls (HC; aged 20 to 55 years; free from diagnosis with brain injury/neurological disorder affecting brain metabolism, and not a first-degree relative to patients with SSD.^15^ All patients had attested ICD-10 (International Classification of Diseases) diagnosis. These two groups were matched for age, sex, and highest educational qualification (see Table 1). Although no formal power analysis was conducted prior to data collection, this sample sizes in fMRI studies with schizophrenia are particularly typical due to recruitment challenges and participants compliance. Importantly, this sample size remains consistent with prior fMRI studies (including our previous studies), and evidence indicates that robust group differences can still be detected.^36–39^ Symptom severity was assessed using the Scale for the Assessment of Positive Symptoms (SAPS) and the Scale for the Assessment of Negative Symptoms (SANS).^40,41^ All patients were considered clinically able to participate and eligible for neuroimaging study. This ability was operationalised as sufficient cognitive and communicative capacity to read, hear, ask and answer questions, understand and provide informed consent, as well as passing a predefined threshold of behavioural task performance. Clinical and demographic information are shown in the Table 1, which has been modified and extended from our previous reports,^24,32^ including scores from symptoms, psychological tests and equivalent doses of antipsychotics.

**Table 1:** Demographic, clinical, and psychomotor task characteristics.

Characteristics	HC (*n* = 20)		SSD (*n* = 20)	
Sex	15 (male)	5 (female)	16 (male)	4 (female)
Attained degree	15 (LS), 5 (US), 2 (PS)		13 (LS), 4 (US), 3 (PS)	
Antipsychotic medication	None		4 (none), 1 (FGA), 15 (SGA)	
Age (Mean ± SD)	38.4 ± 9.9		38.5 ± 8.5	
EHI score	82.8 ± 29.3		86.9 ± 17.5	
MWT-B score	27.1 ± 5.2		27.8 ± 3.9	
d2 score	440.9 ± 85.6		402.8 ± 103.1^+^	
TMT-A (seconds)	31 ± 17		29 ± 7	
TMT-B (seconds)	64 ± 29		80 ± 31	
TMT-(B-A; seconds)	33.45 ± 24.06		**50.45 ± 28.42**	
TMT-(B/A; seconds)	2.26 ± 0.70		2.76 ± 1.08	
WAIS FS score	9.3 ± 1.7		9.7 ± 1.4	
WAIS BS score	8.2 ± 1.7		7.9 ± 1.6	
SPQ-B: CP score	0.9 ± 1.1		**3.8 ± 1.9**	
SAPS total score	1.9 ± 2.4		**12.1 ± 9.8**	
Hallucinations	0.3 ± 0.9		2 ± 4.2	
Delusions	0.4 ± 0.7		**5.6 ± 5.1**	
Delusions of reference	0 ± 0		**1.1 ± 1.2**	
Delusions of being controlled	0 ± 0		**0.5 ± 0.9**	
Residual positive symptoms	1.2 ± 1.2		**4.2 ± 5.1**	
SANS total score	1.3 ± 2.0		**13.6 ± 12.2**	
Affective flattening or blunting	0.10 ± 0.31		**6.60 ± 8.84**	
Alogia	0.15 ± 0.37		**2.15 ± 2.07**	
Avolition or apathy	0.15 ± 0.49		**2.1 ± 2.45**	
Anhedonia or asociality	0.50 ± 0.83		**7.35 ± 6.91**	
Attention	0.20 ± 0.62		**0.05 ± 0.22**	
Olanzapine Equivalent	0 ± 0		**13.36 ± 12.06**	

Note: LS: lower secondary; US: upper secondary; PS: post-secondary; EHI: Edinburgh Handedness Inventory; FGA: first-generation antipsychotics; SGA: second-generation antipsychotics; MWT-B: Mehrfachwahl-Wortschatz-Test (multiple choice vocabulary test); d2:d2 test of attention; TMT: Trail Making Test; WAIS: Wechsler Adult Intelligence Scale; FS: forward span; BS: backward span; SPQ-B: Schizotypal Personality Questionnaire-Brief; CP: Cognitive-Perceptual subscale; SAPS: Scale for the Assessment of Positive Symptoms; SANS: Scale for the Assessment of Negative Symptoms; HC: healthy control; SSD: schizophrenia spectrum disorder. Two patients were medicated with FGA as well as SGA; ^+^missing data for one participant. Values and scores are the mean ± standard deviation. **Bold** values represent significant differences between HC and SSD patients (*p* < 0.05, uncorrected).

The study was approved by the institutional ethics committee from the Philipps University of Marburg. We confirm that all research methods have been performed according to the relevant guidelines in the declaration of Helsinki, all participants provided written informed consent prior to data collection and received financial compensation for their participation.

In addition to antipsychotic medications, a few patients were taking other medications. Benzodiazepines were used by three patients (diazepam, lorazepam). Other medications included antidepressants (*n* = 2; citalopram, escitalopram), mood stabilizers (*n* = 1; valproate), anticholinergic (*n* = 1; akineton), stimulant (*n* = 1; medikinet adult), cardiovascular medications (*n* = 2; bisprolol, HCT: hydrochlorothiazide), and antibiotic (*n* = 1; augmentan). Medication regimens were stable at the time of scanning.

### Task and technical information

An MRI-compatible passive movement device (PMD) was used to perform right-hand movements during the fMRI task. Participants grasped the handle mounted on the PMD, hand movements were performed over approximately 30° arc (~ 5.5 cm trajectory), consisting an extension from left “home” position to the right end and return to the left end. Participants performed active (self-initiated) or passive (generated by PMD via an air pressure controller system) movements. Supplementary Fig. 1 illustrates the hand movement preparation and execution phases modified from previous study.^32^ The following link includes a video demonstration of hand movements with feedback, outside the MRI scanner: 10.5281/zenodo.2621302. Inside the MRI scanner, the PMD was positioned adjacent to the participant’s right thigh.

Real-time hand movement direction and position were recorded through integrated optical fiber sensors in the PMD. Hand movement feedback was captured with a high-speed camera system (MRC High Speed, MRC Systems GmbH, Heidelberg, Germany; frame interval: 4 ms) and projected onto a screen at 60 Hz. This output was visible to participants via a mirror fixed within the MRI head coil. During half of the trials, participants viewed recordings of their hand, in the remaining trials, they observed a pre-recorded hand of an individual with opposite-sex. In addition to an inevitable intrinsic system delay (~ 43 ms), video feedback was displayed with six different delays of 0, 83, 167, 250, 334, and 417 ms, corresponding to frame shifts (0 to 25 frames) at 60 Hz. These delays were used and validated in many of our prior studies, including hand-movements in HC.^34,42^ Task required participants to respond to whether feedback was delayed or not via button presses by left hand. This experimental setup was managed by custom-developed software running on a Windows 7 workstation with specified hardware configurations.

### Design of experiment

This is a mixed-factorial design experiment incorporating two within-subject factors—type of movement execution (active vs. passive) and visual feedback identity (self vs. other hand)—along with a between-subject factor: group (SSD vs. HC). Thus, comprise four movement-feedback conditions: self-active, self-passive, other-active, and other-passive.

### Experimental setup and processes

During the fMRI session, participants lay in a supine position inside the scanner, with the PMD positioned on their right thigh. Subjects were instructed to grip the handle of the PMD using their right hand, grasping the upper part with index finger and thumb and supporting the lower part of the handle with the remaining fingers. Also, was instructed not to move until they are ready to execute movement, feedback was static until movement initiation. Movements involved were extending the hand laterally from left to the right end and then returning it to the original position on the left end. In active blocks, participants were trained to execute each movement within approximately 1500 milliseconds, mirroring the duration of movements in passive conditions. After completing the scanning session, all participants completed a debriefing questionnaire assessing their experience.

The fMRI experiment consisted two separate runs, and each run contained 48 trials, 24 trials per block (active or passive), each block started with an initial cue (“Active” or “Passive”). During active block, participants self-initiated and controlled hand movement preparation and execution. In passive block, participants prepared to relax their hand and let the hand moved by the PMD (controlled by air pressure controller), 500 ms after the video feedback began to match the timing of movement onset across conditions (active vs. passive). Each trial was started with a “Ready” cue and followed by hand’s video feedback (duration: 4000 ms), randomised with either participant’s own hand or a pre-recorded hand (gender-swapped). During movement, participants were asked to detect delays between actual hand movement and displayed video-feedback. Subsequently, participants saw a cue “Delay?” and had to indicate whether they perceived a delay via button press (left middle finger = “yes”; index = “no”). Each trial ended by displaying a black screen, with a randomly jittered inter-trial interval lasting between 2000 and 5000 ms.

### Acquisition of fMRI data

Neuroimaging data were recorded using a 3 Tesla Magnetom Trio Tim MRI system (Siemens, Erlangen, Germany) equipped with a 12-channel head coil. Functional images were collected using a T2*-sensitive gradient-echo echoplanar imaging (EPI) sequence with the following parameters: repetition time (TR) = 1650 ms, echo time (TE) = 25 ms, and a flip angle of 70°. For each of the two runs, 330 volumes were captured in descending order, covering 34 axial slices per volume (matrix size: 64 × 64, field of view: 192 × 192 mm, slice thickness: 4 mm, inter-slice gap: 0.6 mm; resulting voxel size: 3 × 3 × 4.6 mm). High-resolution anatomical scans were acquired using a T1-weighted MPRAGE (Magnetization Prepared Rapid Acquisition Gradient Echo) sequence with a TR of 1900 ms, TE of 2.26 ms, flip angle of 9°, and a matrix resolution of 256 × 256 (field of view: 256 × 256 mm). Each slice was 1 mm thick, resulting in voxel dimensions of 1 × 1 × 1.5 mm, including a 0.5 mm interslice gap.

### Pre-processing of functional MRI data

Functional MRI datasets were preprocessed using standard Statistical Parametric Mapping (SPM12, revision 7771; Wellcome Centre for Human Neuroimaging, University College London), implemented in MATLAB R2021b (MathWorks, Inc.). The pipeline included the following core steps: alignment of functional volumes to correct for head motion, spatial alignment (coregistration) of structural and functional scans, tissue segmentation of the anatomical images, spatial normalisation to the MNI template space with isotropic resampling (2 × 2 × 2 mm³ voxel size), and spatial smoothing using an 8 mm full-width at half-maximum (FWHM) Gaussian kernel. Framewise displacement (FD) metrics were computed to monitor motion artifacts across volumes; less than 10% of volumes showed FD values exceeding 1 mm in any individual run.

### Movement-phase-specific temporal dynamics of BOLD responses

For this study, BOLD responses from the entire movement phase were segmented into their respective preparation and execution phases. To disentangle the contribution of movement intention, phase, and feedback, we employed factorial analyses (movement type [active x passive] x phase [preparation x execution] x feedback [own x other hand]). Passive (driven by the PMD itself) movement was used as a control condition to reflect neural responses without relying on internal initiation (internal agency). This approach is essential for examining disturbances in agency (internal vs. external) mechanisms and the underlying mechanisms of delusional experiences like passivity control.^15,32^ In the first analyses, movement-phase-specific BOLD responses across movement types (active vs. passive), phases (preparation vs. execution), and feedback (own vs. other hand) conditions were modelled by canonical-HRF with TD to detect shift in timing compared to canonical-HRF within a group. In a second analyses, BOLD responses were modelled by canonical-HRF with TD and DD to detect shift in duration compared to canonical-HRF within the group. Delays in the feedback compared to actual hand-movement were not analysed as a separate factor here, because the primary focus of this study was to capture timing and duration of BOLD responses during preparation and execution. During preparation subject were instructed not to move and thus feedback was mainly static, delays were noticeable only during execution with moving hand-feedback.

BOLD response modelling was implemented using the General Linear Model (GLM) framework in SPM12 (https://www.fil.ion.ucl.ac.uk/spm/). In the first level analyses, preprocessed fMRI data were modelled using the GLM. By modelling preparatory and executory phase’s BOLD responses with the canonical-HRF with its TD, subtle shifts in onset timing has been captured, typically in a sub second range ( ~ ± 1), enhancing sensitivity to onset-related neural response timing dynamics.^24,33,34,43^ In the second analysis, by modelling BOLD responses using canonical-HRF with its TD and its DD captured shifts in duration width—i.e., how long neural activity peak is sustained.^24,33,34,43^ When these within group’s results are compared between groups in the group level analyses, differences of shift in timing and duration of neural (BOLD) responses between HC and SSD can be inferred. There is a report that incorporation of more parameters might induce more variations in the parameter estimates, referred to as the bias-variance trade-off. ^44^ It has been reported that adding TD improves sensitivity to shifts in timing and DD to duration changes, while adding canonical-HRF with TD and DD might have subtle effect on the detection sensitivity.^33,35,43^ Accordingly, we conducted two independent analyses to ensure the best approach: first, modelling canonical HRF + TD to assess timing related group differences; second, modelling HRF + TD + DD to detect alterations in response duration.

In first-level analyses, each participant’s model included 8 main task regressors for canonical-HRF, eight for TD, and eight for DD, plus motion parameters and periods for task related cues and questions. Contrast of interest were defined separately for preparation (camera onset to movement onset) and for execution (movement onset to camera offset), across active vs. passive movement and own hand vs. other hand video feedback. Parameter estimates of TD (positive reflects earlier; negative reflects delayed response) and DD (positive reflects shorter; negative reflects prolonged response duration)^24,34^ were extracted and submitted to second level full factorial model for group analyses. Hence, the primary contrasts of interest (e.g., preparation period for active movement with own hand feedback, preparation period for passive movement with own hand feedback) were generated at the first level for each of the 3 regressors per condition (canonical-HRF, TD, DD) and carried forward into group-level full factorial models. Group-wise T- and F-statistics were calculated for canonical-HRF, TD, DD. Time series were high-pass filtered (cut-off: 128 s) to remove low-frequency noise. Furthermore, to ensure the balance in the typically reported Type I and II errors of the fMRI data analyses, Monte Carlo simulations were conducted.^45,46^ The Monte Carlo simulations were employed with 10,000 iterations, resulting a cluster extent threshold of 104 voxels, ensuring correction for multiple comparisons at *P* < 0.05, and voxel level threshold of *P* = 0.005, consistent to previous studies.^15,24,47^ Those clusters that complied with the Monte Carlo-derived cluster threshold were included and interpreted in the results, consistent with the previous study.^15,24,47^ Conjunction and between-group contrasts examined the temporal characteristics in HC and patients with SSD, across both movement preparation and execution phases. Cluster labels were assigned based on the highest percentage of anatomical contribution using atlas AAL 3v1,^48^ and only clusters with at least 50% assignable voxels were included in the results. Coordinates are referenced to the MNI152 standard space.

### Correlation analyses: Timing/Duration vs. symptom severity

Both negative and positive symptoms are associated to dysfunctional self-other differentiability and the predictability of one’s own movement consequences.^11,20,21,49–51^ Positive symptoms were assessed using subscales of SAPS^40^: hallucinations (items 1–7), delusions of reference (item 14), delusions of being controlled (item 15)), and residual positive symptoms (items 21–35). Negative symptoms were assessed using SANS^41^ subscales: affective flattening or blunting (items 1–8), alogia (items 9–13), avolition/apathy (items 14–17), anhedonia/asociality (items 18–22), and attention (items 23–25). Here, we explored correlation between timing of neural responses in different brain areas with scores of these symptoms (Supplementary Table 2). We further assessed specificity of positive symptoms, through performing partial correlation analyses by controlling total SANS score (see Supplementary Table 3). We also explored this partial correlation analyses controlling antipsychotic (olanzapine equivalent) doses along with total SANS score. Some HC individuals have shown a negligible level of psychotic like symptom score. Therefore, we also conducted correlation analyses for HC. All correlations analyses were performed using JASP (Jeffreys’s Amazing Statistics Program; version 0.18.3; JASP team, 2024).

## Results

### Timing (delayed/reversed) of BOLD responses during preparation in SSD

**Fig. 1:**
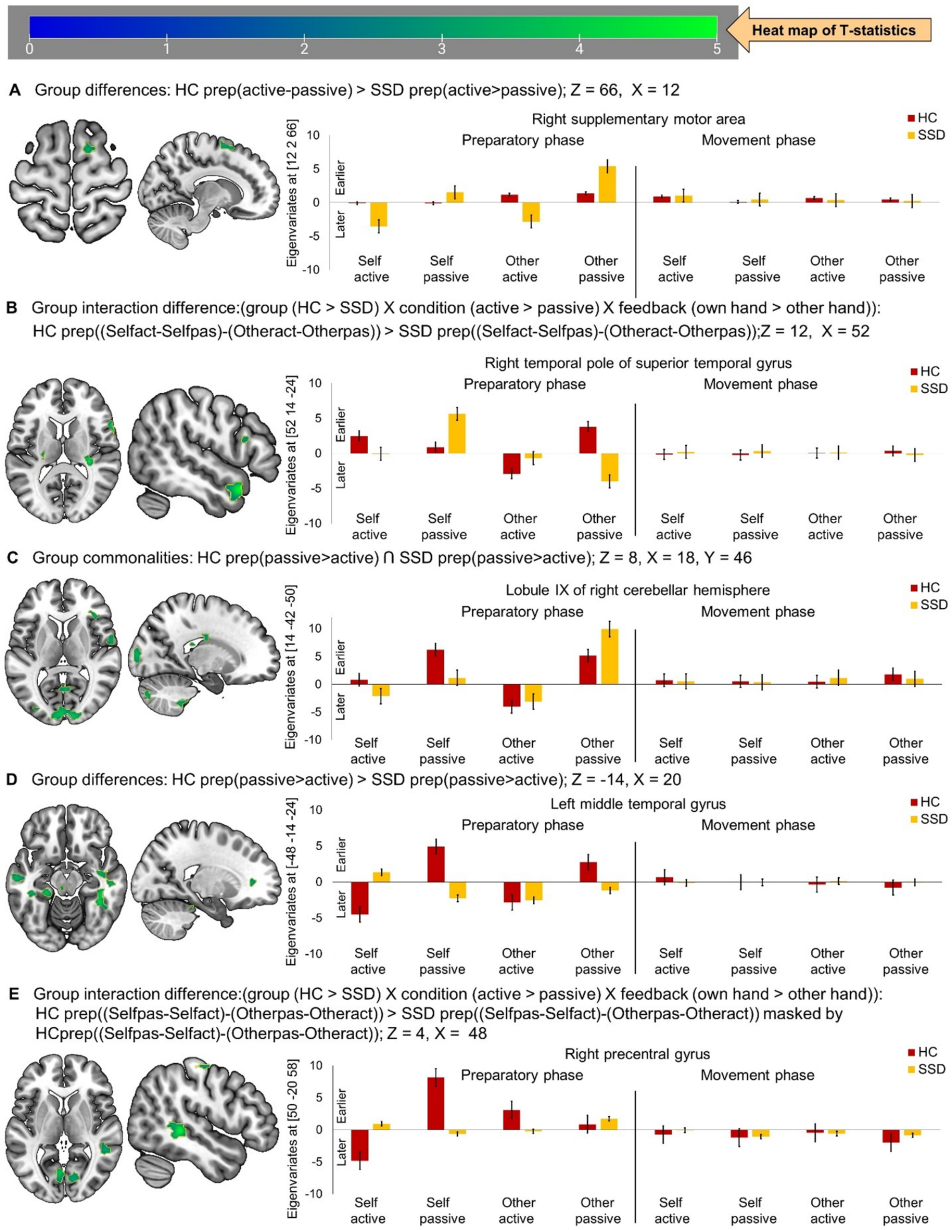
Timing of neural activation in active compared to passive condition and vice versa, during preparation and execution phase. (**A**) Earlier activation specific to HC compared to patients with SSD shown at Z = 66, X = 12; (**B**) Group interaction effect of earlier activation in HC relative to SSD patients shown at Z = 12, X = 52; (**C) **Overlapping activation timing between HC and SSD patients during passive compared to active movement preparation shown at Z = 8, X = 18; (** D**) Differential activation timing between HC over SSD patients during passive compared to active movement preparation shown at Z = -14, X = 20; and (**E**) interaction related earlier activation in HC compared to SSD patients during passive over active movement preparation shown at Z = 4, X = 48. In the bar graph, positive eigenvariates reflect earlier activation, while negative values reflect later activation timing. HC: healthy control, (*n* = 20); SSD: schizophrenia spectrum disorder, (*n* = 20).

Patients with SSD and HC showed no overlapping clusters in BOLD response (neural response) timing for the contrast active > passive hand movement preparation. However, compared to HC, patients with SSD exhibited differences in timing: SSD showed delayed neural responses during active but advanced during passive movement preparation in the right supplementary motor area (Table 2; Fig. 1A). Furthermore, the group interaction analyses (group [HC > SSD] X condition [active > passive] X feedback [own hand > other hand]) revealed clusters mainly comprising the right temporal pole of superior temporal gyrus and right opercular inferior frontal gyrus. When restricting the analyses to brain regions significantly modulated in HC (masked with the interaction HC prep ([Selfact-Selfpas]-[Otheract-Otherpas]), it revealed a cluster mainly comprising the right temporal pole of superior temporal gyrus. In contrast to HC, patients with SSD showed relatively delayed responses during active but advanced during passive movement preparation, especially differentiable with own hand feedback (Table 2; Fig. 1B). Although patterns differed somewhat with other hand feedback, timing alterations in patients were evident across all movement and feedback conditions.

### Timing of BOLD responses: advanced/reversed during preparation in SSD

For the passive > active contrast, patients with SSD were largely comparable to HC in BOLD response timing across clusters comprising mainly the left calcarine fissure and surrounding cortex, crus II of right cerebellar hemisphere, left inferior parietal gyrus, right opercular inferior frontal gyrus, right lingual gyrus, and right rolandic operculum (Supplementary Table 1, Fig. 1C). Conjunction analyses indicated that both groups demonstrated relatively earlier BOLD responses in passive compared to active movement preparation, regardless of feedback modality (Supplementary Table 1). Nevertheless, patients with SSD differed to HC in clusters largely comprising the bilateral middle temporal gyrus, left caudate nucleus, left fusiform gyrus, right postcentral gyrus, left paracentral lobule, and right pregenual anterior cingulate cortex. Specifically, in contrast to HC, patients with SSD exhibited earlier responses during active while delayed during passive movement preparation, this difference was most consistent with own hand feedback (Table 2; Fig. 1D). When this group difference contrast was further masked by active movement preparation with own hand feedback in HC, clusters revealed mainly comprising bilateral middle temporal gyrus, left fusiform gyrus, and right postcentral gyrus (Supplementary Table 1). Here again, patients with SSD most clearly differed during passive movement preparation with own hand feedback, showing delayed response compared to HC (similar to Fig. 1D). Furthermore, the group interaction (group [HC > SSD] X condition [passive> active] X feedback [own hand > other hand]), masked by the interaction of HC (group [HC] X condition [passive> active] X feedback [own hand > other hand]) resulted clusters mainly comprising left superior parietal gyrus, right middle temporal gyrus, right precentral gyrus, and right calcarine fissure and surrounding cortex. In this pattern, HC showed earliest response during passive and latest response during active movement preparation with own feedback, whereas patients with SSD have not shown such preparation specific distinct changes in timing, regardless of the feedback condition (Table 2; Fig. 1E).

**Table 2:** Timing of neural responses during hand movement preparation.

Cluster label	Cluster extends	X	Y	Z	T	Z_E_	k_E_	*P*_FWE−Cluster coorr_.
Group differences: HC prep(active-passive) > SSD prep(active>passive)								
Right supplementary motor area	SMA_R (85.9%), SFG_R (14%)	12	2	66	3.42	3.40	121	0.944
Group interaction difference: HC prep((Selfact-Selfpas)-(Otheract-Otherpas)) > SSD prep((Selfact-Selfpas)-(Otheract-Otherpas))								
Right temporal pole of superior temporal gyrus	TPOsup_R (12%), TPOmid_R (5%), Not assignable (40.7%)	52	14	-24	5.14	5.08	1908	< 0.001
Right inferior frontal gyrus, opercular part	IFGoperc_R (72.1%), IFGtriang_R (16.3%), ROL_R (9.6%)	62	12	12	4.22	4.18	104	0.968
Group interaction: HC prep((Selfact-Selfpas)-(Otheract-Otherpas)) > SSD prep((Selfact-Selfpas)-(Otheract-Otherpas)) masked by HC prep((Selfact-Selfpas)-(Otheract-Otherpas))								
Right temporal pole of superior temporal gyrus	TPOsup_R (52.3%), TPOmid_R (14.7%), MTG_R (11.7%), Not assignable (16.2%)	52	14	-24	5.14	5.08	266	0.552
Group differences: HC prep(passive-active) > SSD prep(passive-active)								
Right middle temporal gyrus	MTG_R (29.6%), FFG_R (11%), ITG_R (9.6%), Not assignable (36.6%)	40	-42	-8	4.71	4.67	1202	0.004
Left caudate nucleus	CAU_L (49.7%), Not assignable (48.3%)	-10	14	12	4.44	4.40	439	0.218
Left fusiform gyrus	FFG_L (25.6%), PHG_L (19.6%), CER_4_5_L (13.2%), CER3_L (9.5%), HIP_L (7.2%), ITG_L (5.4%), Not assignable (18%)	-24	-32	-16	4.14	4.11	484	0.168
Left middle temporal gyrus	MTG_L (71.8%), ITG_L (19.3%), TPOmid_L (5.3%)	-48	-14	-24	4.03	4.00	337	0.387
Left middle temporal gyrus	MTG_L (96.3%)	-62	-48	-6	3.88	3.86	107	0.964
Right postcentral gyrus	PoCG_R (50.8%), PreCG_R (22.7%), IPG_R (18.1%), MFG_R (6.9%)	52	8	48	3.80	3.78	349	0.365
Left paracentral lobule	PCL_L (54.3%), SMA_L (42.1%)	-6	-12	70	3.67	3.65	164	0.854
Right middle temporal gyrus	MTG_R (37.7%), ITG_R (18.9%), HIP_R (5.7%), Not assignable (31.3%)	54	-24	-16	3.58	3.56	297	0.480
Right pregenual anterior cingulate cortex	ACCpre_R (47.3%), Not assignable (48%)	20	38	6	3.30	3.29	129	0.931
Group interaction differences: HC prep((Selfpas-Selfact)-(Otherpas-Otheract)) > SSD prep((Selfpas-Selfact)-(Otherpas-Otheract)) masked by HC prep((Selfpas-Selfact)-(Otherpas-Otheract))								
Left superior parietal gyrus	SPG_L (29.3%), PCUN_L (23.8%), PCUN_R (13.6%), MOG_L (9.9%), SPG_R (7.7%), IPG_L (7.3%)	-10	-68	50	5.37	5.31	1762	< 0.001
Right middle temporal gyrus	MTG_R (76.3%), Not assignable (20.7%)	48	-36	-2	4.84	4.80	232	0.660
Right precentral gyrus	PreCG_R (95.7%)	50	-20	58	4.35	4.32	188	0.789
Right calcarine fissure and surrounding cortex	CAL_R (50.6%), CAL_L (24.7%), LING_L (21%)	-4	-70	6	4.08	4.05	377	0.310

Coordinates are listed in MNI space and used AAL 3v1 cluster labelling. Cluster defining threshold: *p* < 0.005, uncorrected, minimum cluster size = 104 voxels (Monte-Carlo cluster level corrected at *p* < 0.05). Note: FWE-Cluster-corrected values are provided in Table 2. R = right hemisphere; L = left hemisphere. SMA: supplementary motor area; PreCG: precentral gyrus; PoCG: postcentral gyrus; SFG: superior frontal gyrus; MFG: middle frontal gyrus; IFGoperc: inferior frontal gyrus, opercular part; IFGtriang: inferior frontal gyrus, triangular part; PCUN: precuneus; ROL: rolandic operculum; PHG: parahippocampal gyrus; HIP: hippocampus; CAL: calcarine fissure and surrounding cortex; LING: lingual gyrus; CAU: caudate nucleus; MOG: middle occipital gyrus; FFG: fusiform gyrus; SPG: superior parietal gyrus; IPG: inferior parietal gyrus, excluding supramarginal and angular gyri; MTG: middle temporal gyrus; ITG: inferior temporal gyrus; TPOsup: temporal pole, superior temporal gyrus; TPOmid: temporal pole, middle temporal gyrus; PCL: paracentral lobule; CER3: lobule III of cerebellar hemisphere; CER4_5: lobule IV, V of cerebellar hemisphere.

### Duration of BOLD responses: shorten/reversed during preparation in SSD

**Fig. 2:**
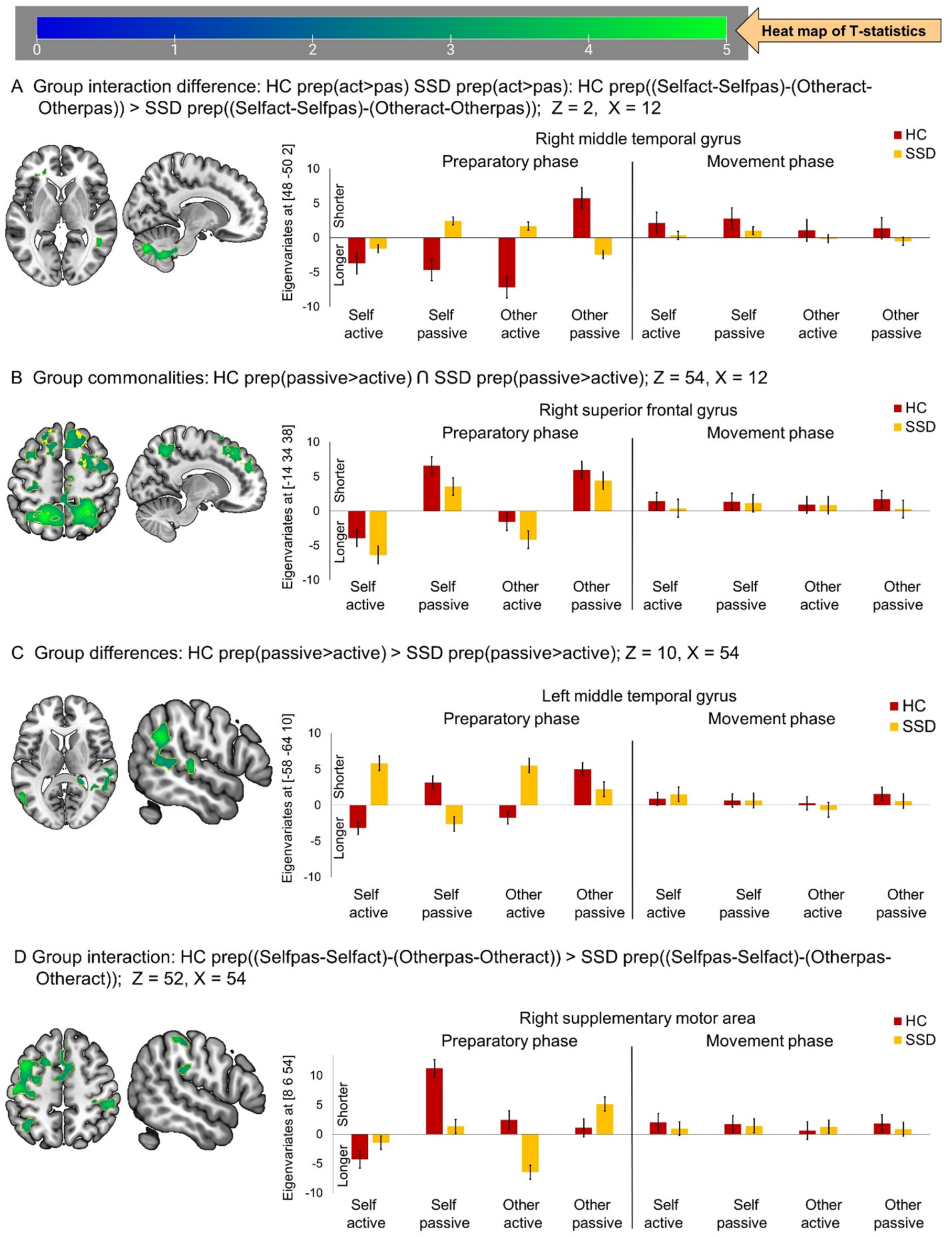
Duration of neural responses during active compared to passive conditions and vice versa, during the preparation and execution phase. (**A**) Group interaction effect of neural response durations in HC relative to SSD patients shown at Z = 2, X = 12; (**B**) Similar duration of neural responses between HC and SSD patients during passive compared to active movement preparation shown at Z = 54, X = 12; (** C**) Differential durations of neural responses in HC over SSD during passive compared to active movement preparation shown at Z = 10, X = 54; (**D**) Group interaction between HC and SSD during passive over active movement preparation shown at Z = 52, X = 54. In the bar graph, positive eigenvariates reflect shorter responses, while negative values reflect longer responses. HC: healthy control, (*n* = 20); SSD: schizophrenia spectrum disorder, (*n* = 20).

In the DD analyses, for the preparation related contrast (active> passive), patients with SSD and HC showed no clusters with overlapping BOLD response durations and no direct group differences. However, group interaction analyses (group [HC > SSD] X condition [active > passive] X feedback [own hand > other hand]) revealed differences in clusters primarily consisting of the crus II of left cerebellar hemisphere and right middle temporal gyrus. Unlike HC, patients with SSD showed shorter BOLD response durations across all conditions, except reversed (longer) during preparation for passive movement with other hand feedback (Table 3; Fig. 2A).

For the passive > active contrast, patients with SSD were broadly similar in clusters involving mainly the left precuneus, right superior frontal gyrus, and left precentral gyrus. Here, in both groups, passive compared to active movement preparation elicited shorter BOLD responses, regardless of hand feedback condition (Table 3; Fig. 2B). The group interaction analyses (group [HC > SSD] X condition [passive> active] X feedback [own hand > other hand]) exhibited clusters mainly comprising the left precentral gyrus, bilateral supramarginal gyrus, right postcentral gyrus, right supplementary motor area, and left inferior parietal gyrus (Table 3; Fig. 2D). In contrast to HC, patients with SSD have not shown the shortest neural responses during passive movement preparation with own hand feedback. However, in group difference analyses, patients with SSD differed in the clusters primarily comprising the bilateral middle temporal gyrus (Table 3; Fig. 2C). In the right middle temporal gyrus, unlike HC, patients with SSD have not shown shorter neural responses during passive and longer during active movement preparation, regardless of feedback condition (Table 3; similar to Fig. 2C). In the left middle temporal gyrus, similar distinct pattern of active (longer) and passive (shorter) movement preparation specific changes in duration of BOLD responses observed in HC; however, this pattern was opposite in patients with SSD (shorter responses during active but longer during passive, most pronounced with own hand feedback; see Table 3; Fig. 2C).

**Table 3:** Duration of neural activation during hand movement preparation.

Cluster label	Cluster extends	X	Y	Z	T	Z_E_	k_E_	*P*_FWE−cluster −coorr_.
Group interaction difference: HC prep((Selfact-Selfpas)-(Otheract-Otherpas)) > SSD prep((Selfact-Selfpas)-(Otheract-Otherpas))								
Crus II of left cerebellar hemisphere	CERCRU2_L (13.4%), CERCRU1_L (13.3%), CER8_R (10.8%), CER9_R (9.3%), CER8_L (9.2%), CERCRU2_R (6.7%), Not assignable (19%)	12	-76	-36	5.14	5.10	3370	< 0.001
Right middle temporal gyrus	MTG_R (68.7%), Not assignable (31.3%)	48	-50	2	4.32	4.30	134	0.911
Group commonalities: HC prep(passive > active) Ո SSD prep(passive > active)								
Left precuneus	PCUN_L (15.9%), PCUN_R (7.3%), ANG_R (11.6%), SPG_L (9.4%), SPG_R (7.7%), IPG_L (8%), IPG_R (7.2%), Not assignable (13.6%)	-20	-56	56	5.27	5.23	5932	< 0.001
Right superior frontal gyrus	SFG_R (22.6%), SFG_L (15.6%), MFG_L (12.7%), MFG_R (10.2%), SFGmed_L (11.4%), SFGmed_R (9.2%)	-14	34	38	4.42	4.40	6190	< 0.001
Left precentral gyrus	PreCG_L (80.5%), PoCG_L (15.3%)	-28	-24	54	3.45	3.44	190	0.753
Group differences: HC prep(passive > active) > SSD prep(passive > active)								
Right middle temporal gyrus	MTG_R (27.9%), STG_R (24.3%), ANG_R (20.2%), SMG_R (10.8%), Not assignable (14%)	54	-50	28	4.72	4.69	1083	0.004
Left middle temporal gyrus	MTG_L (85.3%), MOG_L (11.9%)	-58	-64	10	4.65	4.62	143	0.890
Group interaction: HC prep((Selfpas-Selfact)-(Otherpas-Otheract)) > SSD prep((Selfpas-Selfact)-(Otherpas-Otheract))								
Left precentral gyrus	PreCG_L (39.1%),PoCG_L (27.1%), MFG_L (15.6%), SFG_L (8.6%)	-40	8	52	4.81	4.77	1556	< 0.001
Right supramarginal gyrus	SMG_R (94.7%)	68	-30	30	4.32	4.30	244	0.580
Right postcentral gyrus	PoCG_R (50.4%), IPG_R (41.5%), SMG_R (5.9%)	52	-34	54	4.19	4.17	236	0.605
Left supramarginal gyrus	SMG_L (56.6%), STG_L (26.4%), MTG_L (9.2%), IPG_L (7.2%)	-64	-36	28	4.17	4.15	348	0.314
Right supplementary motor area	SMA_R (43.7%), SMA_L (34.3%), MCC_L (20.4%)	8	6	54	3.83	3.81	545	0.091
Left inferior parietal gyrus	IPG_L (97.4%)	-38	-56	54	3.52	3.51	114	0.950

Coordinates are listed in MNI space and used AAL 3v1 cluster labelling. Cluster defining threshold: *p* < 0.005, uncorrected, minimum cluster size = 104 voxels (Monte-Carlo cluster level corrected at *p* < 0.05). Note: FWE-Cluster-corrected values are provided in Table 3. R = right hemisphere; L = left hemisphere. PreCG: precentral gyrus; SFG: superior frontal gyrus; SFGmed: superior frontal gyrus, medial; MFG: middle frontal gyrus; IFGoperc: inferior frontal gyrus, opercular part; SMG: supra-marginal gyrus; ANG: angular gyrus; PCUN: precuneus; ROL: rolandic operculum; SMA: supplementary motor area; MCC: middle cingulate & paracingulate gyri; HIP: hippocampus; CAL: calcarine fissure and surrounding cortex; CUN: cuneus; PCUN: precuneus; LING: lingual gyrus; MOG: middle occipital gyrus; FFG: fusiform gyrus; PoCG: postcentral gyrus; SPG: superior parietal gyrus; IPG: inferior parietal gyrus, excluding supramarginal and angular gyri; TPOsup: temporal pole, superior temporal gyrus; TPOmid: temporal pole, middle temporal gyrus; STG: superior temporal gyrus; MTG: middle temporal gyrus; PCL: paracentral lobule; CERCRU1: crus I of cerebellar hemisphere; CERCRU2: crus II of cerebellar hemisphere; CER4_5: lobule IV, V of cerebellar hemisphere; CER6: lobule VI of cerebellar hemisphere; CER8: Lobule VIII of cerebellar hemisphere; CER9: lobule IX of cerebellar hemisphere; VER4_5: lobule IV, V of vermis.

### Movement execution phase

During the execution phase, no clusters showed significant differences between active > passive or passive > active contrasts, neither for timing nor for duration of BOLD responses. This absence of group effects is illustrated in the bar plots of Figs. 1 and 2.

### Correlation between timing of neural responses and symptoms

We then explored correlations between movement (active and passive) preparatory neural response timing (compared to HC) and symptom (positive and negative) severity. For passive movement preparation with own hand feedback: advanced timing of neural responses in the right supplementary motor area was negatively correlated to delusions of being controlled and affective flattening or blunting; while delayed timing in the bilateral middle temporal gyrus was negatively correlated to the delusions of being controlled (Supplementary Table 2). No significant correlation emerged for the preparation of active movement with own hand feedback. To isolate symptom specific effect, we further conducted partial correlation analyses controlling for total SANS scores (Supplementary Table 3). Under this control, advanced neural response timing during passive movement preparation with own hand feedback in the right supplementary motor area was negatively correlated to delusions of being controlled; while advanced preparatory neural response timing during active movement with own-hand feedback in the right precentral gyrus was negatively correlated with delusions of being controlled, formal thought disorder, and residual positive symptoms (Supplementary Table 3). Furthermore, these positive symptoms specific partial correlation was even true (significant as shown in supplementary Table 3) when the olanzapine equivalent doses were applied as control along with total SANS score. Regarding response duration, for active movement preparation with own hand feedback, relatively less longer neural responses in the right supplementary motor area was negatively correlated with delusions and alogia; while, for passive movement preparation with own hand feedback, relatively less-shorter neural response was negatively correlated with delusions and anhedonia/asociality. The oppositely shorter neural responses for active movement preparation with own hand feedback in the left middle temporal gyrus was positively correlated to attention (Supplementary Table 4), while right middle temporal gyrus showed no correlation. When controlling negative symptoms, this correlation of duration was not significant. With correction for multiple comparisons, most of these were not significant due to a large number correlations performed. However, for timing, analyses of robustness of significance strength with Spearman’s rank correlation, significance strength was reduced, yet significant particularly when negative symptoms and antipsychotic equivalent doses were controlled. This result may suggest that the original Pearson correlations might have been influenced by extreme values or non-linear relationships.

**Fig. 3:**
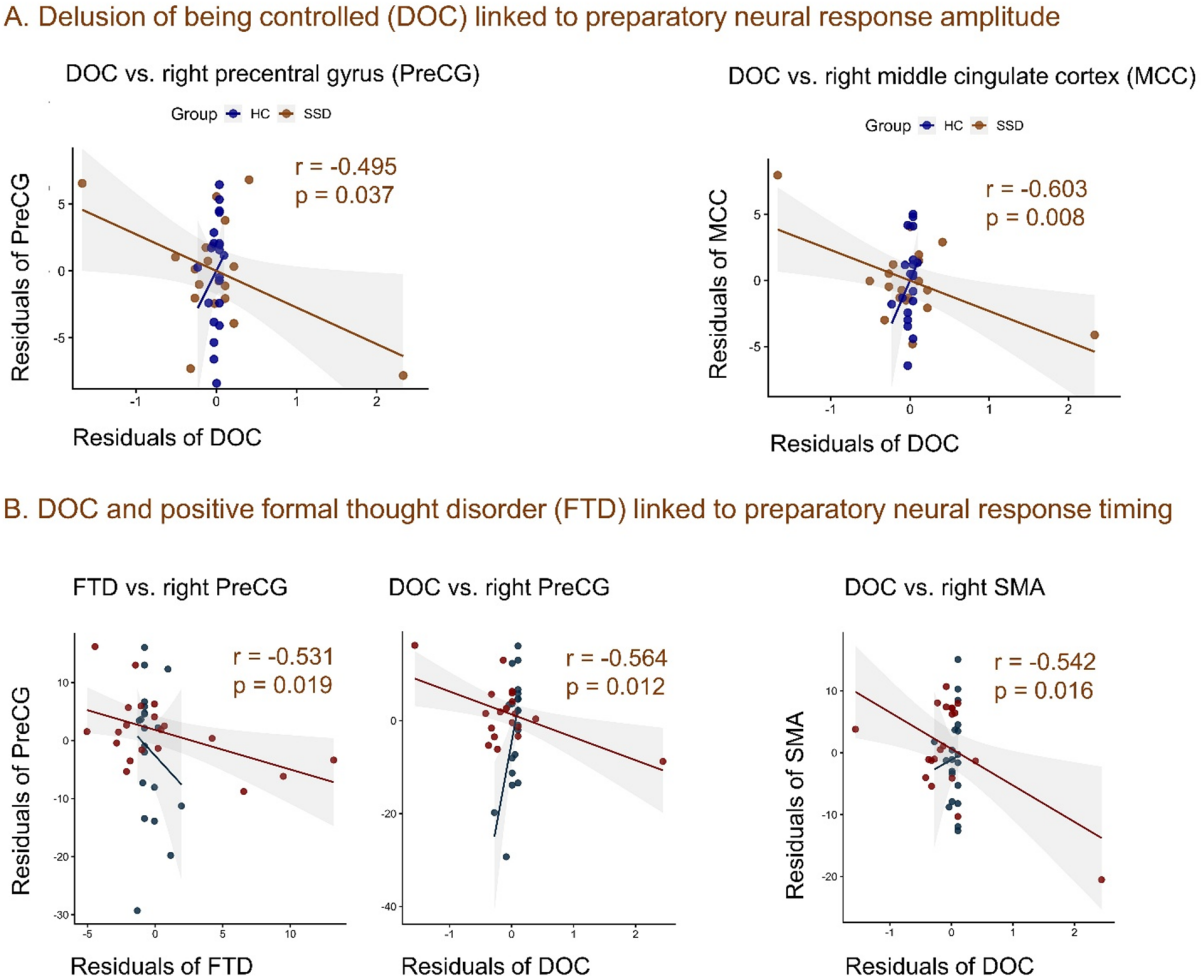
Partial correlations (controlling for negative symptoms, total SANS score) between dysregulated (compared to HC) amplitude and timing of preparatory neural responses under own-hand feedback and positive symptoms in patients with SSD. (**A**) Higher active movement preparatory BOLD amplitudes were positively associated with delusions of being controlled (DOC) in the right precentral gyrus (PreCG), middle cingulate cortex (MCC), and supramarginal gyrus (SMG), indicating dysregulated pre-activation in motor–cognitive integration regions. (**B**) Both DOC and positive formal thought disorder (FTD) were associated with active movement preparatory earlier neural responses (TD effects) in the right PreCG, suggesting altered temporal encoding of motor preparation linked to positive symptoms; DOC was additionally associated with passive movement preparatory earlier neural responses in the right supplementary motor area (SMA), indicating aberrant temporal processing even in externally driven conditions.

The correlation in HC is not significant in any items of SAPS or SANS, the distributions of total SANS and SAPS scores are shown in supplementary Fig. 2. A large number of HC’s scores are near zero to two and mean value is close to zero. Which are consistent with restricted range or floor effect compared to broader score in SSD (2 to 35) (Supplementary Fig. 2). However, these restricted scores are rather consistent with subclinical psychotic symptoms like experiences (e.g., hallucinations or delusions or affective flattening), which are non-distressingly transient and reflect a wide transdiagnostic phenomenon, and may occur at some point of life in any healthy individuals due to a wide range of neurological and psychological situations, but are not episodic or recurrent symptoms.^52–55^.

## Discussion

This fMRI study is the first, to our knowledge, that uncovered movement phase (preparation, execution) specific temporal (timing [TD] and duration [DD]) abnormalities of neural (BOLD) responses in patients with SSD. Findings of this study point to a fundamental disruption in the temporal coordination across the brain—during hand movement, but predominantly during movement preparation compared to execution. This extends our prior study showing amplitude abnormalities predominantly during preparatory phase,^47^ also identified regions across cortical–subcortical–cerebellar networks with timing abnormalities that are predominantly consistent during the entire movement phase.^32^.

Compared to HC, patients with SSD exhibited a striking shift in preparatory response timing patterns across sensory-motor integration areas. Most notably, regardless of feedback conditions, SSD patients exhibited delayed neural responses in the right supplementary motor area during active movement preparation (Table 2; Fig. 1A. In contrast to HC, patients with SSD showed reversed neural response timing profiles for active and passive movement preparation in key sensory-motor areas including the bilateral middle temporal gyrus, left caudate nucleus, left fusiform gyrus, right precentral gyrus, right postcentral gyrus, left paracentral lobule, and right pregenual anterior cingulate cortex—particularly under own hand feedback conditions (Table 2; Fig. 1D and E). Patients with SSD also exhibited atypical patterns in the duration of preparatory neural responses during active and passive movement, diverging from those observed in HCs. Specifically, reversed or diminished modulation in key sensorimotor and psychomotor integration regions, such as the crus II of left cerebellar hemisphere and right middle temporal gyrus (Table 3; Fig. 2A); left precentral gyrus, bilateral supramarginal gyrus, right supplementary motor area, right postcentral gyrus, and left inferior parietal gyrus (Table 3; Fig. 2D); and notably aberrant modulation in the bilateral middle temporal gyrus (Table 3; Fig. 2C). These diminished or reversed modulation of BOLD response timing (Fig. 1) and duration (Fig. 2), underscores a widespread failure of temporal scaffolding across the brain. In this line, studies have been reported abnormal dynamic changes in brain activity across time and complexity of information processing in schizophrenia.^56^ These interpretations are also consistent with disrupted dynamic coordination across cortico-ponto-thalamo-cerebellar regions in SSD.^26,30,31^.

Dysregulated active versus passive movement preparation specific shift in timing, especially, delayed response in supplementary motor area during active and in right precentral gyrus during passive movement was most prominent under own hand-feedback. This aligns with previous report in these regions that active (suppression) and passive (more activation) movement preparation specific activation pattern is reversed in SSD, also reduced regulation or distinction between left vs. right supplementary motor area.^45^ Such amplitude and timing abnormalities in these regions may result in dysregulated volitional control mediated imprecise motor commands and efference copies, leading to impaired sensorimotor outcome prediction, and likely giving imprecise sense of agency over time that have often been reported in SSD.^1,4,57–64^ Our finding of timing and duration abnormalities may resemble deficits of temporal contiguity, neural response synchrony and coordination across cortical, subcortical, and cerebellar networks in schizophrenia.^30,31,63,65,66^ Moreover, disrupted duration dynamics may reflect sensory-motor and psychomotor processing abnormalities reported in SSD—such as psychomotor slowing, impaired frequency, and phase coherence.^1,4,19,23^ This can also be evident in our results from trail making task (TMT) task, because patients with SSD were significantly slower in the TMT task (TMT B-A: shown in Table 1), which may indicate diminished executive psychomotor control and visuo-motor functioning. This interpretation is consistent with commonly reported abnormalities in patients with SSD, for instance, psychomotor functioning (e.g., slowing), sensory-motor feedback integration,^3,13,67–69^ and movement-feedback monitoring.^13,67,70–75^ Taken together, these areas with altered timing and duration might be a broad reflection of disrupted synchrony among motor intention, regulation of preparatory neural activation, cognitive processes related to updating preparatory information, and temporal processing (internal-clock) dysfunction in patients with SSD.^4,32,76–80^.

Anterior cingulate cortex is involved in attention and selection of action types (e.g., active or passive), top-down attentional modulation of sensory processing, a state of preparedness for selecting motor response, and monitoring conflicting response (e.g., active or passive control).^81,82^ In this line, pregenual anterior cingulate cortex has been suggested as the hub connecting attention networks, salience networks, and default mode networks functionality.^83,84^ Moreover, right middle cingulate cortex has shown reversed active versus passive movement preparation specific suppression versus activation pattern, affecting its role in sensorimotor integration and motor control.^47^ Such timing abnormalities found here in right anterior cingulate cortex (Table 2, similar to Fig. 2D) along with previously reported aberrant activation in the right middle cingulate cortex, are in line with their altered functional connectivity to right supplementary motor area, insula, striatum, and thalamus, supporting their role in the impairment of sensorimotor signal integration and motor control experiences (e.g., passivity).^47,84^ While, right middle temporal gyrus has shown reduced variation, this active and passive movement preparation specific shift in neural response timing and duration were reversed in the left middle temporal gyrus (Table 2; Fig. 1D; Table 3; Fig. 2C). Supporting this, aberrant suppression has been reported in the left middle temporal gyrus during preparation.^47^ These abnormalities may reflect desynchronisation or conflict between active versus passive specific semantic and sensorimotor processes during preparation, leading to imprecise ego demarcation. In addition to these timing and duration, amplitude abnormalities has been reported predominantly during preparation phase.^32^ Both of our studies may indicate that imprecise volitional (prospective sense of agency) control is largely the underlying basis of imprecise movement-outcome prediction—and the primary basis of often reported impaired retrospective sense of agency in schizophrenia.^12,14,57,85–87^ Supporting this, studies with self-paced key press with/without tones suggested that schizophrenia is characterised by specific impairments in the prospective sense of agency, the early component of sensory-motor prediction mechanisms.^57,87–89^.

Our previous studies consistently suggested dysfunctions in the insula and putamen, reflected by reduced preparatory pre-activation, ^32^ and delayed neural responses during entire movement phase,^24^ reflecting their critical role in intention with feedback monitoring and causal inference of agency in patients with SSD.^24,90–92^ Consistently, some regions that emerged in preparatory amplitude abnormalities,^32^ also appeared with current preparatory TD (e.g., the right supplementary motor area and right precentral gyrus), and with current DD (e.g., right supplementary motor area and left middle temporal gyrus). However, compared to entire hand movement phased TD,^24^ several key regions did not appear in this separated preparatory TD or DD (e.g., left thalamus, left insula, left putamen, and lobule VIII of right cerebellum). This might mean that neural response timing in these regions is not different during preparation alone, but only when time windows include also the execution phase, most likely relevant to continuous sensory-motor feedback integration and causal inference of the sense of agency. Considering altered timing during the whole movement process^24^ and aberrant preparatory amplitudes in SSD patients^32^; this further could mean that earlier effects in those areas likely reflect a stable deficit in preparatory BOLD amplitudes, while timing throughout the whole movement period, but not during isolated preparatory timing or duration dynamics. In contrast, the current approach, separating preparation from movement and by applying the TD and DD analyses—uncovered additional regions with altered timing or duration that were not previously detected,^24,47^ such as left caudate nucleus and right (opercular inferior frontal gyrus, superior frontal gyrus, superior temporal pole of superior temporal gyrus, pregenual anterior cingulate cortex). These novel findings highlight movement phase specific distinct neural signatures—some regions reflect reduced preparatory amplitudes, some aberrant timing in preparatory phase, while others demonstrate disorganised temporal scaffolding during entire movement phase.

The supplementary motor area plays a central role in motor planning, interhemispheric coordination, and inhibitory control during movement preparation.^4,93,94^ Psychomotor processing abnormalities have been consistently implicated in in SSD.^4^ Notably, reduced activation in the left and hyperactivation in the right supplementary motor areas and right precentral gyrus in SSD have been reported,^32^ reflecting disrupted motor planning and coordination deficits during preparation. Our current findings extend these observations, showing consistent (three dimensional) abnormalities in amplitude, timing, and duration of BOLD responses, particularly in the right supplementary motor area and right precentral gyrus during active and passive movement preparation with own hand feedback. Reduced preparatory attenuation have been reported in the left (medial superior frontal gyrus, supplementary motor area, supramarginal gyrus, and superior parietal gyrus). These disruptions likely reflect the role of frontal regions, supplementary motor area, and parietal areas in imprecise motor planning, deficient inhibitory control, and impaired coordination mechanisms in patients with SSD. Supporting this, prior studies have shown pervasive inhibitory control deficits—spanning motor, cognitive, and attentional processes across several tasks in SSD patients.^5–7,95^ Importantly, the supplementary motor area also directly interacts with the precentral gyrus, postcentral gyrus, thalamus, and posterior parietal areas—forming predictive and sensory-motor integrative systems, essential for goal-directed behaviour.^96–98^ The precentral gyrus is further implicated in higher-order cognitive processes, including attention, movement intention selection, movement organisation-imagery, coordination, and inhibition of unwanted movements.^60^.

We found that abnormalities in preparatory amplitude^47^ and timing of neural responses in several regions of the right hemisphere were specifically linked to positive symptoms (Fig. 3A). Notably, advanced preparatory activity in the right supplementary motor area during passive under own-hand feedback correlated negatively with delusions of being controlled (Supplementary Table 3, Fig. 3B). Conversely, earlier preparatory activity in the right precentral gyrus during active with own-hand feedback was negatively associated with delusions of being controlled, formal thought disorder (Supplementary Table 3, Fig. 3B). Additionally, delayed passive movement preparatory activity in the bilateral middle temporal gyrus was also linked to delusions of being controlled, although not when controlling for negative symptoms. Furthermore, shorter preparatory BOLD responses in the right supplementary motor area during active movement was negatively correlated with delusions and alogia, while the duration of passive movement was correlated with delusions and anhedonia/asociality. Conversely, prolonged preparatory duration in the left middle temporal gyrus during active movement was positively correlated with attention—although many of these associations were not sustained after correction for multiple comparisons. Although, not confirmatory, these exploratory moderate Pearson and Spearman’s correlation coefficients—particularly in the right supplementary motor area, right precentral gyrus, and bilateral middle temporal gyrus—underscores potential links between amplitude and temporally dysregulated preparatory neural responses to formal thought disorder and delusions of being controlled (imprecise predictive mechanisms and disrupted prospective sense of agency) in schizophrenia (Fig. 3). These interpretations are consistent with EEG studies with thumb movement and finger pressing, reported abnormalities (timing, duration, and amplitude) in the RP and LRP (mostly around supplementary motor area and precentral gyrus) that occurred before movement onset, and their negative correlation to higher positive and negative symptoms in schizophrenia.^21,22,99,100^ These delayed and reduced or abolished RP and LRP were further correlated with and implicated in ego-disturbances, including self-awareness and delusions of being controlled.^20,32^.

Previous studies reported diminished or inversed temporal lobe (left > right) asymmetry and brain activity was further implicated in formal thought disorder and ego-disturbances (e.g., passivity,^11,12,49,101^ which is consistent with our preparatory amplitude^47^ and timing abnormalities in the middle temporal gyrus, temporal pole of superior temporal gyrus, and cingulate cortex. In the same line, structural (volume, thickness) and functional (metabolic and BOLD responses) abnormalities in broad temporal lobe, specifically left temporal gyrus, right anterior cingulate cortex have also been reported as neural correlate linked to thought disorder in schizophrenia.^101,102^ Furthermore, a meta-analyses have similarly highlighted the role of the supplementary motor area in the sense of agency—where the left middle temporal gyrus, right superior temporal gyrus, and left inferior parietal gyrus activation have been linked to sense of agency attribution, especially in negative agency (reduced motor control).^103^.

Taken together, our findings suggest that examining the temporal dynamics of neural responses not only characterises the neural underpinnings of sense of agency but also offers three-dimensional (amplitude, timing, duration) translational insights into the sense of movement agency dysfunctions in schizophrenia. These findings open a new research avenue into movement phase specific neural response timing and duration, alongside amplitude, as critical dimensions of brain function. Beyond schizophrenia, disturbances in the sense of agency, movement preparation, and sensorimotor integration have also been consistently reported across neurological and psychiatric conditions, including stroke, functional neurological disorder, and depression.^8,103–106^ Therefore, future studies in larger samples should examine movement phase specific shared and distinct temporal dynamics across these disorders and enable the transfer of mechanistic insights. Such a transdiagnostic approach may ultimately advance the development of neuromechanistically informed neurotechnological interventions (e.g., non-invasive brain stimulations^107^ aimed at restoring the sense of agency, improving motor-sensory function, and self-awareness.

### Limitations

While the current study offers novel insights into the temporal dynamics of preparatory neural activity in SSD patients, a few limitations should be acknowledged. First, the relatively small sample size (*n* = 20 per group) limits the generalisability of the correlation analyses. The observed associations between neural response timing/duration and symptom severity—although compelling, future studies should be extended to a larger cohort. Although Pearson correlation coefficients were moderate to large, the application of stringent statistical correction procedures (e.g., family-wise error correction and correction for multiple comparisons) resulted a lack of significance, while the application of more robust Spearman’s rank correlation yielded moderate correlation coefficients. Second, although TD and DD models offer improved sensitivity to detect changes in neural response timing and duration, these analyses are inherently limited by the temporal resolution of fMRI, its indirect measure of neural responses, and the assumptions of the canonical HRF. However, fMRI is still one of the most advanced and suitable imaging methods for the whole brain spatiotemporal dynamics. Finally, most patients (*n* = 15) were taking second-generation antipsychotics (SGAs), one was taking first-generation antipsychotics (FGA), and the remaining four were not taking antipsychotics but other medications. We may not rule out a minor impact of antipsychotics on neural response timing, but our results are robust. To ensure potential medication effects, additional analyses was conducted focusing on olanzapine equivalent dose. The repeated-measure ANOVA within key regions (right supplementary motor area and right precentral gyrus) did not reveal any significant effects of medication dosage as covariate on neural response timing. Additionally, a sensitivity analysis excluding the four unmedicated patients was performed, and the pattern of results remained unchanged. Thus, it is unlikely that antipsychotic dose can explain such robust result pattern. Furthermore, it has further been reported that motor and agency abnormalities exist in drug-naive schizophrenia and across patients with psychosis, first-episode schizophrenia, neurological, and neurodegenerative disorders, not using antipsychotics.^3,108,109^ Taken together, these findings suggest that the observed timing abnormalities are unlikely to be primarily driven by medication effects.

## Conclusion

This is the first fMRI study analysing movement type (active versus passive) and their phase (preparation, execution) specific neural activity (BOLD) with TD and DD, identified shift in timing and duration of neural responses across cortical, subcortical, and cerebellar regions. Findings indicated that patients with SSD differed from HCs primarily in the preparatory phase, but not during movement execution. Our findings demonstrate aberrant temporal dynamics of neural responses in SSD, characterised by deviation in timing and duration during active and passive hand movement preparation. These characteristic patterns may reflect deficits in the prospective component of the sense of agency during predictive processing in schizophrenia. The distinct timing and duration pattern across cortical and subcortical regions may indicate that temporal synchronisation between areas is impaired during movement preparation and across the entire movement phase. Such disruptions affect the integration of internal and external sensory-motor signals, compromising the coordination needed for movement control and sense of agency. Notably, the distinction between active and passive movement specific shift in neural responses were diminished or reversed. Additionally, dysregulated preparatory neural response timing of right hemispheric regions was linked with positive symptom severity, notably the right supplementary motor area and right precentral gyrus, which may reflect impaired preparatory ego-demarcation awareness (active versus passive) movement, and disrupted coordination between cortical and subcortical neural activity. These three-dimensional (amplitude, timing, and duration) abnormalities in neural mechanisms may underlie the breakdown of predictive mechanisms, disturbed sense of agency, and ego-disturbances in patients with SSD. Therefore, the incorporation of temporal characteristics alongside amplitude of BOLD responses is very promising to obtain meaningful additional features of underlying neural processes in patients and ultimately characterise impairments in clinical populations.

## Supplementary Information

Below is the link to the electronic supplementary material.

Supplementary Material 1

## Data Availability

The data and scripts supporting this study’s findings can be found in this link: https://doi.org/10.5281/zenodo.15806253. The corresponding author will be the contact for any queries.

## References

[1] Lefebvre, S. et al. The pathobiology of psychomotor slowing in psychosis: altered cortical excitability and connectivity. *Brain* **147**, 1423–1435 (2024). 38537253 10.1093/brain/awad395 PMC10994557

[2] Mikanmaa, E. et al. Towards a neurodynamical understanding of the prodrome in schizophrenia. *NeuroImage* **190**, 144–153 (2019). 29175199 10.1016/j.neuroimage.2017.11.026

[3] Walther, S. et al. Movement disorder and sensorimotor abnormalities in schizophrenia and other psychoses - European consensus on assessment and perspectives. *Eur. Neuropsychopharmacol.* **38**, 25–39 (2020). 32713718 10.1016/j.euroneuro.2020.07.003

[4] Walther, S. et al. Psychomotor Slowing in Psychosis and Inhibitory Repetitive Transcranial Magnetic Stimulation: A Randomized Clinical Trial. *JAMA Psychiatry*. **81**, 563 (2024). 38416468 10.1001/jamapsychiatry.2024.0026 PMC10902782

[5] Gotra, M. Y. et al. Distinguishing patterns of impairment on inhibitory control and general cognitive ability among bipolar with and without psychosis, schizophrenia, and schizoaffective disorder. *Schizophr Res.* **223**, 148–157 (2020). 32674921 10.1016/j.schres.2020.06.033 PMC7704797

[6] Eskridge, C. L. M. et al. Deficits in generalized cognitive ability, visual sensorimotor function, and inhibitory control represent discrete domains of neurobehavioral deficit in psychotic disorders. *Schizophr Res.* **236**, 54–60 (2021). 34392106 10.1016/j.schres.2021.07.036 PMC8464494

[7] Michael, G. A. et al. Attention in schizophrenia: Impaired inhibitory control, faulty attentional resources, or both? *Psychiatry Res.* **290**, 113164 (2020). 32521381 10.1016/j.psychres.2020.113164

[8] Mastria, G. et al. Body ownership alterations in stroke emerge from reduced proprioceptive precision and damage to the frontoparietal network. *Med* **6**, 100536 (2025). 39532102 10.1016/j.medj.2024.10.013

[9] Perez, D. L. et al. Decade of progress in motor functional neurological disorder: continuing the momentum. (2020). 10.1136/jnnp-2020-323953 PMC8440656 33722822

[10] Stangeland, H., Orgeta, V. & Bell, V. Poststroke psychosis: a systematic review. *J. Neurol. Neurosurg. Psychiatry*. **89**, 879–885 (2018). 29332009 10.1136/jnnp-2017-317327 PMC6204934

[11] Leube, D., Whitney, C. & Kircher, T. The neural correlates of ego-disturbances (passivity phenomena) and formal thought disorder in schizophrenia. *Eur. Arch. Psychiatry Clin. Neurosci.* **258** (Suppl 5), 22–27 (2008). 18985290 10.1007/s00406-008-5017-z

[12] Synofzik, M., Thier, P., Leube, D. T., Schlotterbeck, P. & Lindner, A. Misattributions of agency in schizophrenia are based on imprecise predictions about the sensory consequences of one’s actions. *Brain* **133**, 262–271 (2010). 19995870 10.1093/brain/awp291

[13] van der Weiden, A., Prikken, M. & van Haren, N. E. M. Self–other integration and distinction in schizophrenia: A theoretical analysis and a review of the evidence. *Neurosci. Biobehav Rev.* **57**, 220–237 (2015). 26365106 10.1016/j.neubiorev.2015.09.004

[14] Kircher, T. T. J. & Leube, D. T. Self-consciousness, self-agency, and schizophrenia. *Conscious. Cogn.* **12**, 656–669 (2003). 14656508 10.1016/s1053-8100(03)00071-0

[15] Uhlmann, L., Pazen, M., Van Kemenade, B. M., Kircher, T. & Straube, B. Neural Correlates of Self-other Distinction in Patients with Schizophrenia Spectrum Disorders: The Roles of Agency and Hand Identity. *Schizophr Bull.* **47**, 1399–1408 (2021). 33433625 10.1093/schbul/sbaa186 PMC8379550

[16] Miyawaki, Y., Otani, T. & Morioka, S. Impaired Relationship between Sense of Agency and Prediction Error Due to Post-Stroke Sensorimotor Deficits. *J. Clin. Med.* **11**, 3307 (2022). 35743378 10.3390/jcm11123307 PMC9225153

[17] Uhlhaas, P. J. & Singer, W. Abnormal neural oscillations and synchrony in schizophrenia. *Nat. Rev. Neurosci.* **11**, 100–113 (2010). 20087360 10.1038/nrn2774

[18] Northoff, G. & Gomez-Pilar, J. Overcoming Rest–Task Divide—Abnormal Temporospatial Dynamics and Its Cognition in Schizophrenia. *Schizophr Bull.* **47**, 751–765 (2021). 33305324 10.1093/schbul/sbaa178 PMC8661394

[19] Wolff, A. et al. It’s in the timing: reduced temporal precision in neural activity of schizophrenia. *Cereb. Cortex*. **32**, 3441–3456 (2022). 34875019 10.1093/cercor/bhab425 PMC9376870

[20] Donati, F. L. et al. Reduced readiness potential and post-movement beta synchronization reflect self-disorders in early course schizophrenia. *Sci. Rep.* **11**, 15044 (2021). 34294767 10.1038/s41598-021-94356-5 PMC8298598

[21] Vöckel, J. et al. Movement initiation and preparation in subjects with schizophrenia - The amplitude of the readiness potential as a biological marker for negative symptom severity. *Schizophr Res.* **260**, 3–11 (2023). 37543008 10.1016/j.schres.2023.07.012

[22] Karaman, T., Özkaynak, S., Yaltkaya, K. & Büyükberker, Ç. Bereitschaftpotential in schizophrenia. *Br. J. Psychiatry*. **171**, 31–34 (1997). 9328491 10.1192/bjp.171.1.31

[23] Wolff, A. & Northoff, G. Temporal imprecision of phase coherence in schizophrenia and psychosis—dynamic mechanisms and diagnostic marker. *Mol. Psychiatry*. **29**, 425–438 (2024). 38228893 10.1038/s41380-023-02337-z

[24] Rashid HA, Kircher T, Straube B. Temporal characteristics of hemodynamic responses during active and passive hand movements in schizophrenia spectrum disorder. Schizophrenia (Heidelb). 2025 Aug 6;11(1):109. doi: 10.1038/s41537-025-00654-6. PMID: 40770189; PMCID: PMC12328563. 10.1038/s41537-025-00654-6 PMC12328563 40770189

[25] Palesi, F. et al. Contralateral cortico-ponto-cerebellar pathways reconstruction in humans in vivo: implications for reciprocal cerebro-cerebellar structural connectivity in motor and non-motor areas. *Sci. Rep.* **7**, 12841 (2017). 28993670 10.1038/s41598-017-13079-8 PMC5634467

[26] Ha, M. et al. Aberrant cortico-thalamo-cerebellar network interactions and their association with impaired cognitive functioning in patients with schizophrenia. *Schizophrenia* **9**, 50 (2023). 37573437 10.1038/s41537-023-00375-8 PMC10423253

[27] Welniarz, Q., Worbe, Y. & Gallea, C. The Forward Model: A Unifying Theory for the Role of the Cerebellum in Motor Control and Sense of Agency. *Front. Syst. Neurosci.* **15**, 644059 (2021). 33935660 10.3389/fnsys.2021.644059 PMC8082178

[28] Woodward, N. D. Thalamocortical Functional Connectivity, Cognitive Impairment, and Cognitive Remediation in Schizophrenia. *Biol. Psychiatry Cogn. Neurosci. Neuroimaging*. **2**, 307–309 (2017). 29560919 10.1016/j.bpsc.2017.03.013

[29] Giraldo-Chica, M., Rogers, B. P., Damon, S. M., Landman, B. A. & Woodward, N. D. Prefrontal-Thalamic Anatomical Connectivity and Executive Cognitive Function in Schizophrenia. *Biol. Psychiatry*. **83**, 509–517 (2018). 29113642 10.1016/j.biopsych.2017.09.022 PMC5809301

[30] Abram, S. V., Hua, J. P. Y. & Ford, J. M. Consider the pons: bridging the gap on sensory prediction abnormalities in schizophrenia. *Trends Neurosci.* **45**, 798–808 (2022). 36123224 10.1016/j.tins.2022.08.008 PMC9588719

[31] Abram, S. V. et al. Pons-to-Cerebellum Hypoconnectivity Along the Psychosis Spectrum and Associations With Sensory Prediction and Hallucinations in Schizophrenia. *Biol. Psychiatry Cogn. Neurosci. Neuroimaging*. **9**, 693–702 (2024). 38311290 10.1016/j.bpsc.2024.01.010 PMC11227403

[32] Rashid, H. A., Kircher, T. & Straube, B. Aberrant preparation of hand movement in schizophrenia spectrum disorder: An fMRI study. https://doi.org/10.1093/braincomms/fcaf148 (2025). 10.1093/braincomms/fcaf148 10.1093/braincomms/fcaf148 PMC12022610 40290422

[33] Friston, K. J. et al. *Event-Related fMRI: Characterizing Differ. Responses NeuroImage* **7**, 30–40 (1998). 9500830 10.1006/nimg.1997.0306

[34] Kavroulakis, E., van Kemenade, B. M., Arikan, B. E., Kircher, T. & Straube, B. The effect of self-generated versus externally generated actions on timing, duration, and amplitude of blood oxygen level dependent response for visual feedback processing. *Hum. Brain Mapp.* **43**, 4954–4969 (2022). 36056611 10.1002/hbm.26053 PMC9582366

[35] Hopfinger, J. B., Büchel, C., Holmes, A. P. & Friston, K. J. A Study of Analysis Parameters That Influence the Sensitivity of Event-Related fMRI Analyses. *NeuroImage* **11**, 326–333 (2000). 10725188 10.1006/nimg.2000.0549

[36] Straube, B., Green, A., Sass, K. & Kircher, T. Superior Temporal Sulcus Disconnectivity During Processing of Metaphoric Gestures in Schizophrenia. *Schizophr Bull.* **40**, 936–944 (2014). 23956120 10.1093/schbul/sbt110 PMC4059440

[37] Straube, B., Wroblewski, A., Jansen, A. & He, Y. The connectivity signature of co-speech gesture integration: The superior temporal sulcus modulates connectivity between areas related to visual gesture and auditory speech processing. *NeuroImage* **181**, 539–549 (2018). 30025854 10.1016/j.neuroimage.2018.07.037

[38] Streiling, K., Schülke, R., Straube, B. & Van Dam, L. C. J. Choice- and trial-history effects on causality perception in Schizophrenia Spectrum Disorder. *Schizophrenia* **11**, 65 (2025). 40246862 10.1038/s41537-025-00614-0 PMC12006371

[39] Wroblewski, A., He, Y. & Straube, B. Dynamic Causal Modelling suggests impaired effective connectivity in patients with schizophrenia spectrum disorders during gesture-speech integration. *Schizophr Res.* **216**, 175–183 (2020). 31882274 10.1016/j.schres.2019.12.005

[40] Andreasen, N. C. & University of Iowa. Department of Psychiatry. Scale for the assessment of positive symptons: (SAPS). (1984).

[41] Scale for the Assessment of Negative Symptoms. (SANS)*. *Br. J. Psychiatry*. **155**, 53–58 (1989). 2695141

[42] Uhlmann, L. et al. Seeing your own or someone else’s hand moving in accordance with your action: The neural interaction of agency and hand identity. *Hum. Brain Mapp.* **41**, 2474–2489 (2020). 32090439 10.1002/hbm.24958 PMC7268012

[43] Henson, R. N. A., Price, C. J., Rugg, M. D., Turner, R. & Friston, K. J. Detecting latency differences in event-related BOLD responses: application to words versus nonwords and initial versus repeated face presentations. *NeuroImage* **15**, 83–97 (2002). 11771976 10.1006/nimg.2001.0940

[44] Poldrack, R. A., Mumford, J. A. & Nichols, T. E. *Handbook of Functional MRI Data Analysis* (Cambridge University Press, 2011). 10.1017/CBO9780511895029

[45] Lieberman, M. D. & Cunningham, W. A. Type I and Type II error concerns in fMRI research: re-balancing the scale. *Soc. Cogn. Affect. Neurosci.* **4**, 423–428 (2009). 20035017 10.1093/scan/nsp052 PMC2799956

[46] Slotnick, S. D. Cluster success: fMRI inferences for spatial extent have acceptable false-positive rates. *Cogn. Neurosci.* **8**, 150–155 (2017). 28403749 10.1080/17588928.2017.1319350

[47] Rashid, H. A., Kircher, T. & Straube, B. Aberrant preparation of hand movement in schizophrenia spectrum disorder: An fMRI study. Preprint at (2025). 10.1093/braincomms/fcaf148 10.1093/braincomms/fcaf148 PMC12022610 40290422

[48] Rolls, E. T., Huang, C. C., Lin, C. P., Feng, J. & Joliot, M. Automated anatomical labelling atlas 3. *NeuroImage* 206, 116189 (2020). 10.1016/j.neuroimage.2019.116189 31521825

[49] Fuchs, T. From self-disorders to ego disorders. *Psychopathology* **48**, 324–331 (2015). 26345993 10.1159/000432404

[50] Frith, C. The self in action: Lessons from delusions of control. *Conscious. Cogn.* **14**, 752–770 (2005). 16098765 10.1016/j.concog.2005.04.002

[51] Strauss, G. P., Bartolomeo, L. A. & Harvey, P. D. Avolition as the core negative symptom in schizophrenia: relevance to pharmacological treatment development. *Npj Schizophr*. **7**, 16 (2021). 33637748 10.1038/s41537-021-00145-4 PMC7910596

[52] Staines, L. et al. Psychotic experiences in the general population, a review; definition, risk factors, outcomes and interventions. *Psychol. Med.* **52**, 3297–3308 (2022). 36004805 10.1017/S0033291722002550 PMC9772919

[53] Rejek, M. & Misiak, B. Dimensions of psychopathology associated with psychotic-like experiences: Findings from the network analysis in a nonclinical sample. *Eur. Psychiatry*. **66**, e56 (2023). 37439195 10.1192/j.eurpsy.2023.2429 PMC10486255

[54] Misiak, B., Pytel, A. & Stańczykiewicz, B. A systematic review of studies using network analysis to assess dynamics of psychotic-like experiences in community samples. *Psychol. Med.* **55**, e54 (2025). 39967317 10.1017/S0033291725000261 PMC12080667

[55] Van Os, J. & Reininghaus, U. Psychosis as a transdiagnostic and extended phenotype in the general population. *World Psychiatry*. **15**, 118–124 (2016). 27265696 10.1002/wps.20310 PMC4911787

[56] Liu, L. et al. Neuroimaging markers of aberrant brain activity and treatment response in schizophrenia patients based on brain complexity. *Transl Psychiatry*. **14**, 365 (2024). 39251595 10.1038/s41398-024-03067-8 PMC11384759

[57] Voss, M. et al. Altered awareness of action in schizophrenia: a specific deficit in predicting action consequences. *Brain* **133**, 3104–3112 (2010). 20685805 10.1093/brain/awq152

[58] Voss, M., Ingram, J. N., Haggard, P. & Wolpert, D. M. Sensorimotor attenuation by central motor command signals in the absence of movement. *Nat. Neurosci.* **9**, 26–27 (2006). 16311591 10.1038/nn1592 PMC2636578

[59] Haggard, P. & Whitford, B. Supplementary motor area provides an efferent signal for sensory suppression. *Cogn. Brain Res.* **19**, 52–58 (2004). 10.1016/j.cogbrainres.2003.10.018 14972358

[60] Bhattacharjee, S. et al. The role of primary motor cortex: more than movement execution. *J. Mot Behav.* **53**, 258–274 (2021). 32194004 10.1080/00222895.2020.1738992

[61] Frith, C. D., Blakemore, S. J. & Wolpert, D. M. Abnormalities in the awareness and control of action. *Philos. Trans. R Soc. Lond. B Biol. Sci.* **355**, 1771–1788 (2000). 11205340 10.1098/rstb.2000.0734 PMC1692910

[62] Moore, J. W., Ruge, D., Wenke, D., Rothwell, J. & Haggard, P. Disrupting the experience of control in the human brain: pre-supplementary motor area contributes to the sense of agency. *Proc. R Soc. B Biol. Sci.* **277**, 2503–2509 (2010). 10.1098/rspb.2010.0404 PMC2894930 20375048

[63] Beño-Ruiz-de-la-Sierra, R. M. et al. Corollary Discharge Dysfunction as a Possible Substrate of Anomalous Self-experiences in Schizophrenia. *Schizophr Bull.* **sbad157**10.1093/schbul/sbad157 (2023). 10.1093/schbul/sbad157 PMC11349017 37951230

[64] Li, S., Zhu, H. & Tian, X. Corollary discharge versus efference copy: distinct neural signals in speech preparation differentially modulate auditory responses. *Cereb. Cortex*. **30**, 5806–5820 (2020). 32542347 10.1093/cercor/bhaa154

[65] J Roth, M. et al. Impaired perception of temporal contiguity between action and effect is associated with disorders of agency in schizophrenia. *Proc. Natl. Acad. Sci.* **120**, e2214327120 (2023). 37186822 10.1073/pnas.2214327120 PMC10214164

[66] Ford, J. M. & Mathalon, D. H. Efference Copy, Corollary Discharge, Predictive Coding, and Psychosis. *Biol. Psychiatry Cogn. Neurosci. Neuroimaging*. **4**, 764–767 (2019). 31495399 10.1016/j.bpsc.2019.07.005

[67] Bansal, S., Murthy, K. G., Fitzgerald, J., Schwartz, B. L. & Joiner, W. M. Reduced transfer of visuomotor adaptation is associated with aberrant sense of agency in schizophrenia. *Neuroscience* **413**, 108–122 (2019). 31228588 10.1016/j.neuroscience.2019.06.012 PMC6687512

[68] Machado, S. et al. Sensorimotor integration: basic concepts, abnormalities related to movement disorders and sensorimotor training-induced cortical reorganization. *Rev Neurol*. 20859923

[69] Silverstein, S. M. & Lai, A. The Phenomenology and Neurobiology of Visual Distortions and Hallucinations in Schizophrenia: An Update. *Front. Psychiatry*. **12**, 684720 (2021). 34177665 10.3389/fpsyt.2021.684720 PMC8226016

[70] Yaple, Z. A., Tolomeo, S. & Yu, R. Abnormal prediction error processing in schizophrenia and depression. *Hum. Brain Mapp.* **42**, 3547–3560 (2021). 33955106 10.1002/hbm.25453 PMC8249895

[71] Stegmayer, K. et al. Limbic interference during social action planning in schizophrenia. *Schizophr Bull.* **44**, 359–368 (2018). 28575506 10.1093/schbul/sbx059 PMC5814975

[72] Hoonakker, M., Doignon-Camus, N. & Bonnefond, A. Sustaining attention to simple visual tasks: a central deficit in schizophrenia? A systematic review. *Ann. N Y Acad. Sci.* **1408**, 32–45 (2017). 29090832 10.1111/nyas.13514

[73] Kircher, T. T. J. & Leube, D. T. Self-consciousness, self-agency, and schizophrenia. *Conscious. Cogn.* **12**, 656–669 (2003). 14656508 10.1016/s1053-8100(03)00071-0

[74] Frith, C. D. & Done, D. J. Experiences of alien control in schizophrenia reflect a disorder in the central monitoring of action. *Psychol. Med.* **19**, 359–363 (1989). 2762440 10.1017/s003329170001240x

[75] Klein, T. A., Ullsperger, M. & Danielmeier, C. Error awareness and the insula: links to neurological and psychiatric diseases. *Front Hum. Neurosci* **7**, (2013). 10.3389/fnhum.2013.00014 PMC3563042 23382714

[76] Osborne, K. J., Walther, S., Shankman, S. A. & Mittal, V. A. Psychomotor slowing in Schizophrenia: Implications for endophenotype and biomarker development. *Biomark. Neuropsychiatry*. **2**, 100016 (2020). 33738459 10.1016/j.bionps.2020.100016 PMC7963400

[77] Osborne, K. J. et al. Timing dysfunction and cerebellar resting state functional connectivity abnormalities in youth at clinical high-risk for psychosis. *Psychol. Med.* **51**, 1289–1298 (2021). 32008594 10.1017/S0033291719004161 PMC9754787

[78] Thuaire, F., Rondepierre, F., Vallet, G. T., Jalenques, I. & Izaute, M. Executive deficits in schizophrenia: mediation by processing speed and its relationships with aging. *Psychol. Med.* **52**, 1126–1134 (2022). 32840193 10.1017/S0033291720002871

[79] Thuaire, F. et al. Executive functions in schizophrenia aging: Differential effects of age within specific executive functions. *Cortex* **125**, 109–121 (2020). 31981891 10.1016/j.cortex.2019.12.003

[80] Dutschke, L. L. et al. Gesture impairments in schizophrenia are linked to increased movement and prolonged motor planning and execution. *Schizophr Res.* **200**, 42–49 (2018). 28709771 10.1016/j.schres.2017.07.012

[81] Adams, R. & David, A. S. Patterns of anterior cingulate activation in schizophrenia: a selective review. *Neuropsychiatr Dis. Treat.* **3**, 87–101 (2007). 19300540 10.2147/nedt.2007.3.1.87 PMC2654525

[82] Crottaz-Herbette, S. & Menon, V. Where and When the Anterior Cingulate Cortex Modulates Attentional Response: Combined fMRI and ERP Evidence. *J. Cogn. Neurosci.* **18**, 766–780 (2006). 16768376 10.1162/jocn.2006.18.5.766

[83] Wang, X. et al. Anterior insular cortex plays a critical role in interoceptive attention. *eLife* **8**, e42265 (2019). 30985277 10.7554/eLife.42265 PMC6488299

[84] Wang, D. et al. Altered functional connectivity of the cingulate subregions in schizophrenia. *Transl Psychiatry*. **5**, e575–e575 (2015). 26035059 10.1038/tp.2015.69 PMC4490280

[85] Synofzik, M., Vosgerau, G. & Voss, M. The experience of agency: an interplay between prediction and postdiction. *Front. Psychol.* **4**, 127 (2013). 23508565 10.3389/fpsyg.2013.00127 PMC3597983

[86] Lindner, A., Thier, P., Kircher, T. T. J., Haarmeier, T. & Leube, D. T. Disorders of Agency in Schizophrenia Correlate with an Inability to Compensate for the Sensory Consequences of Actions. *Curr. Biol.* **15**, 1119–1124 (2005). 15964277 10.1016/j.cub.2005.05.049

[87] Voss, M., Chambon, V., Wenke, D., Kühn, S. & Haggard, P. In and out of control: brain mechanisms linking fluency of action selection to self-agency in patients with schizophrenia. *Brain* **140**, 2226–2239 (2017). 28899009 10.1093/brain/awx136

[88] Haggard, P. Sense of agency in the human brain. *Nat. Rev. Neurosci.* **18**, 196–207 (2017). 28251993 10.1038/nrn.2017.14

[89] Haggard, P., Clark, S. & Kalogeras, J. Voluntary action and conscious awareness. *Nat. Neurosci.* **5**, 382–385 (2002). 11896397 10.1038/nn827

[90] Gebhardt, S. & Nasrallah, H. A. The role of the insula in cognitive impairment of schizophrenia. *Schizophr Res. Cogn.* **32**, 100277 (2023). 36654887 10.1016/j.scog.2022.100277 PMC9841050

[91] Tinaz, S. et al. Insula as the Interface Between Body Awareness and Movement: A Neurofeedback-Guided Kinesthetic Motor Imagery Study in Parkinson’s Disease. *Front. Hum. Neurosci.* **12**, 496 (2018). 30581383 10.3389/fnhum.2018.00496 PMC6292989

[92] Scalabrini, A., Wolman, A. & Northoff, G. The self and its right insula—differential topography and dynamic of right vs. left insula. *Brain Sci.* **11**, 1312 (2021). 34679377 10.3390/brainsci11101312 PMC8533814

[93] Mayer, A. R. et al. Evidence for asymmetric inhibitory activity during motor planning phases of sensorimotor synchronization. *Cortex* **129**, 314–328 (2020). 32554227 10.1016/j.cortex.2020.04.028 PMC7390684

[94] Welniarz, Q. et al. The supplementary motor area modulates interhemispheric interactions during movement preparation. *Hum. Brain Mapp.* **40**, 2125–2142 (2019). 30653778 10.1002/hbm.24512 PMC6865634

[95] Ethridge, L. E. et al. Behavioral response inhibition in psychotic disorders: Diagnostic specificity, familiality and relation to generalized cognitive deficit. *Schizophr Res.* **159**, 491–498 (2014). 25261042 10.1016/j.schres.2014.08.025 PMC4253557

[96] Edwards, L. L., King, E. M., Buetefisch, C. M. & Borich, M. R. Putting the Sensory into sensorimotor control: the role of sensorimotor integration in goal-directed hand movements after stroke. *Front. Integr. Neurosci.* **13**, 16 (2019). 31191265 10.3389/fnint.2019.00016 PMC6539545

[97] Rahimpour, S., Rajkumar, S. & Hallett, M. The Supplementary Motor Complex in Parkinson’s Disease. (2022). 10.14802/jmd.21075 PMC8820882 34814237

[98] Rurak, B. K. et al. SMA-M1 connectivity in older than younger adults measured using dual‐site TMS. *Eur. J. Neurosci.* **54**, 6533–6552 (2021). 34470079 10.1111/ejn.15438

[99] Kappenman, E. S. et al. Electrophysiological Evidence for Impaired Control of Motor Output in Schizophrenia. *Cereb. Cortex*. **26**, 1891–1899 (2016). 25618891 10.1093/cercor/bhu329 PMC6169463

[100] Dreher, J. C. et al. Planning dysfunction in schizophrenia: impairment of potentials preceding fixed/free and single/sequence of self-initiated finger movements. *Exp. Brain Res.* **124**, 200–214 (1999). 9928843 10.1007/s002210050615

[101] Sharkey, R. J. et al. Differences in the neural correlates of schizophrenia with positive and negative formal thought disorder in patients with schizophrenia in the ENIGMA dataset. *Mol. Psychiatry*. **29**, 3086–3096 (2024). 38671214 10.1038/s41380-024-02563-z PMC11449795

[102] Kaur, A. et al. Structural and functional alterations of the temporal lobe in schizophrenia: A Literature Review. *Cureus*10.7759/cureus.11177 (2020). 33262914 10.7759/cureus.11177 PMC7689947

[103] Zito, G. A., Wiest, R. & Aybek, S. Neural correlates of sense of agency in motor control: A neuroimaging meta-analysis. *PLOS ONE*. **15**, e0234321 (2020). 32502189 10.1371/journal.pone.0234321 PMC7274441

[104] Nahab, F. B., Kundu, P., Maurer, C., Shen, Q. & Hallett, M. Impaired sense of agency in functional movement disorders: An fMRI study. *PLOS ONE*. **12**, e0172502 (2017). 28448504 10.1371/journal.pone.0172502 PMC5407819

[105] McCombe Waller, S. et al. Impaired motor preparation and execution during standing reach in people with chronic stroke. *Neurosci. Lett.* **630**, 38–44 (2016). 27436481 10.1016/j.neulet.2016.07.010

[106] Perez, D. L., Keshavan, M. S., Scharf, J. M., Boes, A. D. & Price, B. H. Bridging the great divide: what can neurology learn from psychiatry? *J. Neuropsychiatry Clin. Neurosci.* **30**, 271–278 (2018). 29939105 10.1176/appi.neuropsych.17100200 PMC6309772

[107] Bühler, J., Weber, S., Loukas, S., Walther, S. & Aybek, S. Non-invasive neuromodulation of the right temporoparietal junction using theta-burst stimulation in functional neurological disorder. *BMJ Neurol. Open.* **6**, e000525 (2024). 38361967 10.1136/bmjno-2023-000525 PMC10868242

[108] Perju-Dumbrava, L. & Kempster, P. Movement disorders in psychiatric patients. *BMJ Neurol. Open.* **2**, e000057 (2020). 33681793 10.1136/bmjno-2020-000057 PMC7871724

[109] Waddington, J. L. Psychosis in Parkinson’s disease and parkinsonism in antipsychotic-naive schizophrenia spectrum psychosis: clinical, nosological and pathobiological challenges. *Acta Pharmacol. Sin*. **41**, 464–470 (2020). 32139896 10.1038/s41401-020-0373-y PMC7470778

