# Supplementary material for: Disrupted neural response timing and duration during hand movement preparation in schizophrenia spectrum disorder: An fMRI study
Source: Sci Rep. 2026 May 2;16:14041. doi: 10.1038/s41598-026-50969-2 (PMC13135506; doi:10.1038/s41598-026-50969-2)
Supplement: Supplementary file 1 — Supplementary Material 1 [file 41598_2026_50969_MOESM1_ESM.docx]

Supplementary material for the manuscript: Disrupted neural response timing and duration during hand movement preparation in schizophrenia spectrum disorder: An fMRI study


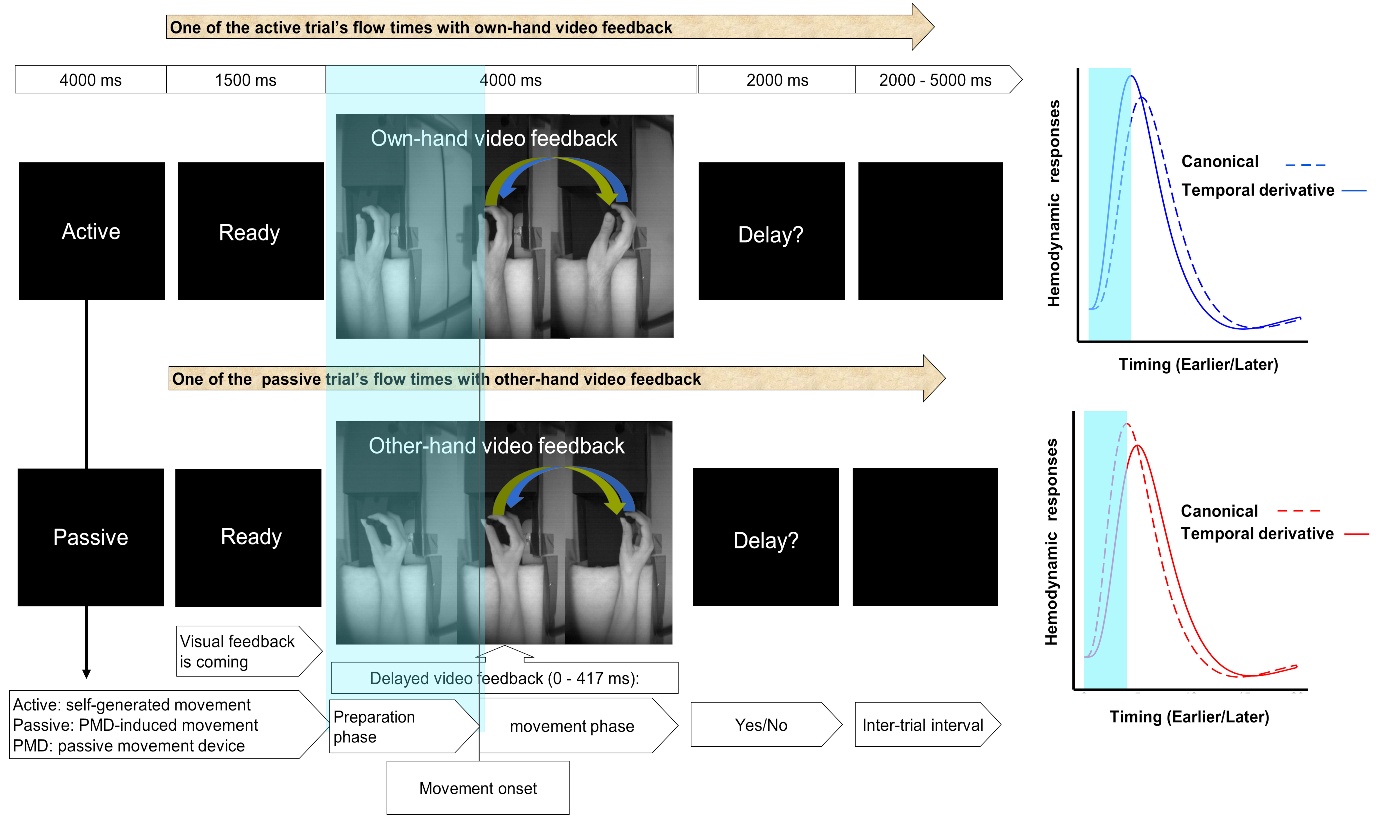


**Supplementary Figure 1: Hand movement preparation and execution phase.** A video demonstration of the hand movement with video feedback is available at the link: <http://doi.org/10.5281/zenodo.2621302>. As shown in the video, there were two blocks: the block for self-initiated (active) movement began with a cue “Active”, and the block for passive movement device (PMD; passive) movement with a cue “Passive”. At the start of each run, participants either moved their hand when cued “Active” (24 trials), or relaxed while the PMD moved it during “Passive” (24 trials). Each trial began with a “Ready” cue, followed by visual feedback for movement and a final “Delay?” question. A black screen (2000–5000 ms) appeared during the inter-trial interval. During the preparation, the right-hand feedback (1st hand; marked in light blue square) remained static until the subject started moving. The movement execution began from left (2nd hand) to right end (3rd hand), and subsequently returned to the left (2^nd^ hand). For male participants, the top row shows active trials with own-hand video; the bottom row shows passive trials with other-hand (pre-recorded image from the female). For female participants, the other-hand videos showed a pre-recorded male hand. Self/other-hand feedback was presented randomly across trials. On the right, expected ideal canonical-hemodynamic response function (HRF) and canonical-HRF with its temporal derivative curves for active and passive movements are shown, which are expected to vary during the preparation and during the execution period.


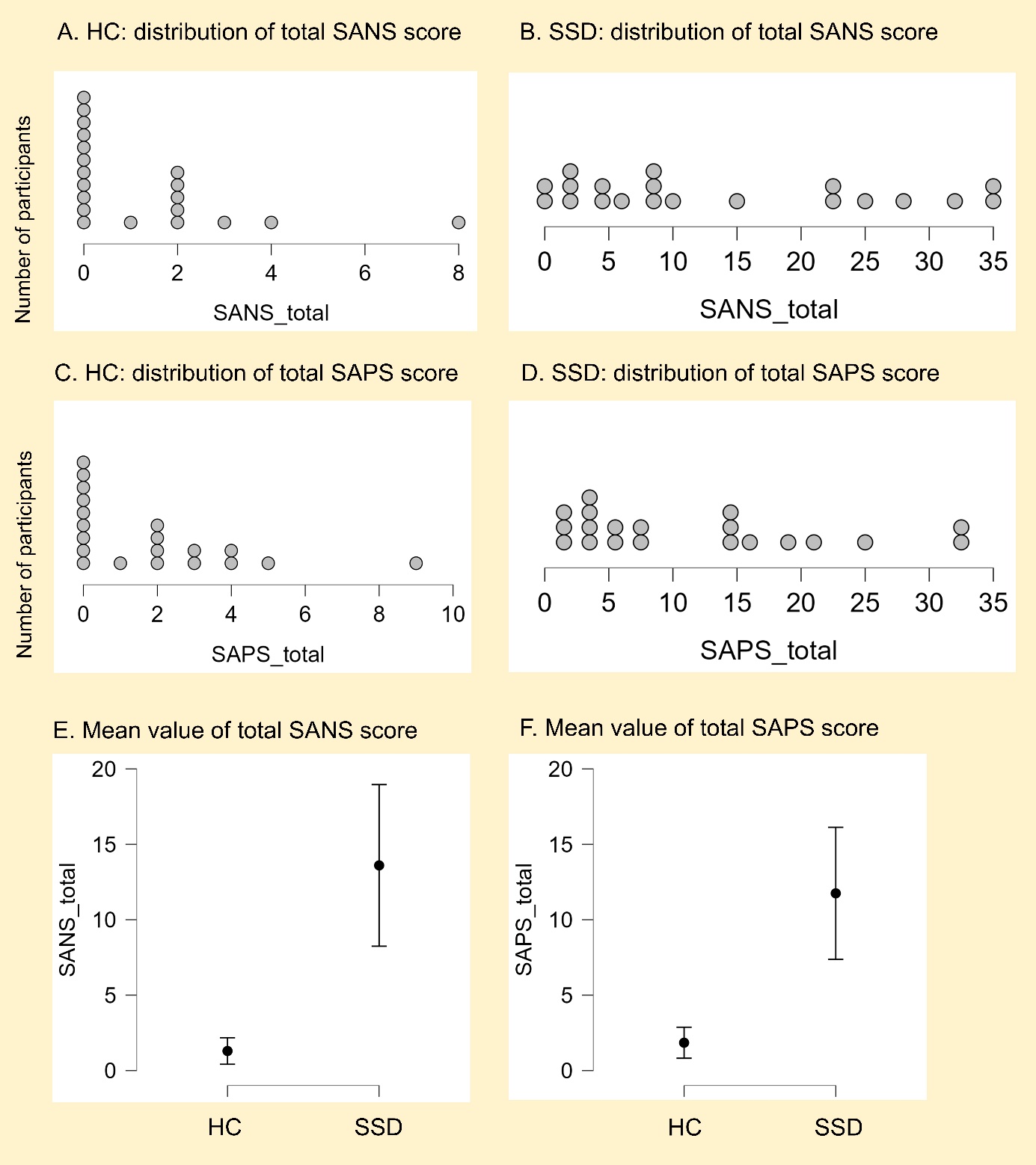


Supplementary figure 2: Distribution of SAPS (scale for the assessment of positive) and SANS (scale for the assessment of negative symptoms) in HC (n = 20; healthy control [A, C]) and in SSD (n = 20; schizophrenia spectrum disorders [B, D]). Mean scores by groups are shown in figure E for total SANS and F for total SAPS score.

Supplementary Table 1: Results regarding activation timing

| Cluster label | Cluster extends | X | Y | Z | T | Z_E_ | k_E_ | P_FWC-coorr._ |
| --- | --- | --- | --- | --- | --- | --- | --- | --- |
| **Group commonalities: HC prep(passive>active) Ո SSD prep(passive>active)** | | | | | | | | |
| Left calcarine fissure and surrounding cortex | CAL_L (16.8%), LING_L (15.8%), MOG_L (9.5%), IOG_L (9.1%), CAL_R (8.5%), FFG_L (8.2%), CERCRU1_L (7.1%), | 12 | -94 | 4 | 4.21 | 4.18 | 1898 | <0.001 |
| Crus II of right cerebellar hemisphere | CERCRU2_R (71.7%), CERCRU1_R (25%) | 12 | -82 | -38 | 4.11 | 4.08 | 244 | 0.625 |
| Left inferior parietal gyrus | IPG_L (52.4%), SPG_L (33.8%), Not assignable (12.4%) | -38 | -56 | 60 | 4.09 | 4.06 | 275 | 0.538 |
| Left supplementary motor area | SMA_L (65.8%), SMA_R (11.7%), SFGmed_R (10.2%), SFGmed_L (6.6%), SFG_L (5.1%), | 0 | 10 | 66 | 4.03 | 4.00 | 196 | 0.766 |
| Right inferior frontal gyrus, opercular part | IFGoperc_R (45.7%), IFGtriang_R (41.5%), Not assignable (10.4%) | 36 | 16 | 30 | 3.95 | 3.92 | 164 | 0.854 |
| Lobule IX of right cerebellar hemisphere | CER9_R (80.8%), Not assignable (16.4%) | 14 | -42 | -50 | 3.85 | 3.83 | 104 | 0.968 |
| Right lingual gyrus | LING_R (50.2%), LING_L (21.6%), VER_4_5 (11.9%), CAL_L (5.2%), Not assignable (8.4%) | 6 | -64 | 2 | 3.71 | 3.69 | 631 | 0.073 |
| Right Rolandic operculum | ROL_R (30.7%), PoCG_R (25.9%), STG_R (24.4%), SMG_R (11.9%), | 56 | -6 | 12 | 3.53 | 3.51 | 352 | 0.357 |
| **Group differences: HC prep(passive-active) > SSD prep(passive-active) masked by HCselfpas** | | | | | | | | |
| Right middle temporal gyrus | MTG_R (28.1%), FFG_R (11.9%), ITG_R (10.4%), Not assignable (37.8%) | 40 | -42 | -8 | 4.71 | 4.67 | 1111 | 0.006 |
| Left fusiform gyrus | FFG_L (36.1%), PHG_L (13.1%), CER_4_5_L (21.9%), CER3_L (12.2%), HIP_L (7.2%), ITG_L (8.3%), Not assignable (8.3%) | -24 | -32 | -16 | 4.14 | 4.11 | 288 | 0.503 |
| Left middle temporal gyrus | MTG_L (77.9%), ITG_L (18.1%) | -48 | -14 | -24 | 4.03 | 4.00 | 249 | 0.611 |
| Right postcentral gyrus | PoCG_R (43.6%), PreCG_R (26.1%), IPG_R (20.8%), MFG_R (7.9%) | 52 | 8 | 48 | 3.80 | 3.78 | 303 | 0.465 |
| Right middle temporal gyrus | MTG_R (41.6%), ITG_R (16.9%), Not assignable (29.4%) | 54 | -24 | -16 | 3.58 | 3.56 | 255 | 0.593 |

Coordinates are listed in MNI space and used AAL 3v1 cluster labelling. Cluster defining threshold: *p* < 0.005, uncorrected, minimum cluster size = 104 voxels (Monte-Carlo cluster level corrected at *p* < 0.05). (Note: FWE-Cluster-corrected values are provided in Table2); R = right hemisphere; L = left hemisphere. PreCG: precentral gyrus; PoCG: postcentral gyrus; SFG: superior frontal gyrus; MFG: middle frontal gyrus; SFGmed: Superior frontal gyrus, medial; IFGoperc: inferior frontal gyrus, opercular part; IFGtriang: Inferior frontal gyrus, triangular part; SMG: supra-marginal gyrus; PCUN: precuneus; ROL: Rolandic operculum; ACCpre: pregenual anterior cingulate cortex; PHG: Parahippocampal gyrus; HIP: hippocampus; CAL: Calcarine fissure and surrounding cortex; LING: lingual gyrus; MOG: middle occipital gyrus; IOG: Inferior occipital gyrus; FFG: fusiform gyrus; SPG: superior parietal gyrus; IPG: inferior parietal gyrus, excluding supramarginal and angular gyri; MTG: middle temporal gyrus; ITG: inferior temporal gyrus; TPOsup: temporal pole, superior temporal gyrus; TPOmid: temporal pole, middle temporal gyrus; CERCRU1: crus I of cerebellar hemisphere; CERCRU2: crus II of cerebellar hemisphere; CER3: Lobule III of cerebellar hemisphere; CER4_5: lobule IV, V of cerebellar hemisphere; CER9: lobule IX of cerebellar hemisphere; VER4_5: lobule IV, V of vermis.

Supplementary Table 2. Correlation between neural activation timing (clusters’ eigenvariates) and symptom scores in SSD patients

| Cluster consisting mainly | symptom | Pearson correlation coeficient: r | p | Effect size (Fisher’s z) | Spearman correlation coeficient: rho  correlation strength: r/rho^1^  0.00–0.10 Negligible  0.10–0.39 Weak  0.40–0.69 Moderate  0.70–0.89 Strong  0.90–1.00 Very strong |
| --- | --- | --- | --- | --- | --- |
| Right supplementary motor area  Preparation of passive movement with own hand feedback | SAPS_I | -0.294 | 0.209 | -0.303 | -0.038 |
|  | SAPS_14 | -0.051 | 0.832 | -0.051 | 0.028 |
|  | SAPS_15 | -0.480 | **0.032** | -0.523 | 0.017 |
|  | SAPS_II | -0.038 | 0.874 | -0.038 | 0.030 |
|  | SAPS_III | 0.146 | 0.538 | 0.147 | -0.051 |
|  | SAPS_IV | -0.066 | 0.782 | -0.066 | 0.272 |
|  | SAPS_res | -0.001 | 0.996 | -0.001 | 0.137 |
|  | SANS_I | -0.463 | **0.040** | -0.501 | -0.209 |
|  | SANS_II | 0.304 | 0.192 | 0.314 | 0.242 |
|  | SANS_III | -0.299 | 0.200 | -0.308 | -0.014 |
|  | SANS_IV | -0.024 | 0.919 | -0.024 | 0.011 |
|  | SANS_V | -0.069 | 0.773 | -0.069 | -0.179 |
|  | SANS_total | -0.125 | 0.599 | -0.126 | -0.069 |
| Left middle temporal gyrus  Preparation of passive movement with own hand feedback | SAPS_I | -0.400 | 0.081 | -0.423 | -0.110 |
|  | SAPS_14 | -0.051 | 0.830 | -0.051 | 0.053 |
|  | SAPS_15 | -0.500 | **0.025** | -0.549 | -0.137 |
|  | SAPS_II | -0.208 | 0.378 | -0.211 | -0.142 |
|  | SAPS_III | 0.248 | 0.292 | 0.253 | 0.181 |
|  | SAPS_IV | 0.039 | 0.869 | 0.039 | 0.098 |
|  | SAPS_res | 0.137 | 0.564 | 0.138 | 0.176 |
|  | SANS_I | -0.401 | 0.080 | -0.425 | -0.080 |
|  | SANS_II | -0.006 | 0.979 | -0.006 | -0.087 |
|  | SANS_III | -0.341 | 0.141 | -0.358 | -0.051 |
|  | SANS_IV | -0.172 | 0.469 | -0.173 | -0.158 |
|  | SANS_V | -0.186 | 0.432 | -0.188 | -0.219 |
|  | SANS_total | -0.308 | 0.186 | -0.318 | -0.247 |
| Right middle temporal gyrus  Preparation of passive movement with own hand feedback | SAPS_I | -0.334 | 0.150 | -0.348 | -0.312 |
|  | SAPS_14 | -0.179 | 0.450 | -0.181 | -0.097 |
|  | SAPS_15 | -0.517 | **0.019** | -0.573 | -0.205 |
|  | SAPS_II | -0.240 | 0.308 | -0.245 | -0.184 |
|  | SAPS_III | -0.064 | 0.790 | -0.064 | -0.208 |
|  | SAPS_IV | -0.393 | 0.086 | -0.416 | -0.208 |
|  | SAPS_res | -0.388 | 0.091 | -0.409 | -0.325 |
|  | SANS_I | -0.207 | 0.382 | -0.210 | -0.002 |
|  | SANS_II | -0.076 | 0.749 | -0.077 | -0.177 |
|  | SANS_III | -0.400 | 0.089 | -0.423 | -0.209 |
|  | SANS_IV | -0.207 | 0.381 | -0.210 | -0.157 |
|  | SANS_V | 0.237 | 0.314 | 0.242 | 0.259 |
|  | SANS_total | -0.321 | 0.167 | -0.333 | -0.256 |
| Right precentral gyrus  Preparation of active movement with own hand feedback | SAPS_I | 0.100 | 0.676 | 0.100 | 0.289 |
|  | SAPS_14 | -0.230 | 0.329 | -0.234 | -0.134 |
|  | SAPS_15 | -0.168 | 0.480 | -0.169 | 0.076 |
|  | SAPS_II | 0.343 | 0.139 | 0.357 | 0.186 |
|  | SAPS_III | 0.063 | 0.792 | 0.063 | 0.070 |
|  | SAPS_IV | -0.407 | 0.075 | -0.432 | -0.354 |
|  | SAPS_res | -0.349 | 0.131 | -0.364 | -0.323 |
|  | SANS_I | -0.229 | 0.331 | -0.233 | -0.259 |
|  | SANS_II | 0.550 | **0.012** | 0.618 | 0.333 |
|  | SANS_III | -0.011 | 0.964 | -0.011 | 0.044 |
|  | SANS_IV | 0.362 | 0.116 | 0.380 | 0.182 |
|  | SANS_V | -0.033 | 0.891 | -0.033 | -0.020 |
|  | SANS_total | 0.329 | 0.156 | 0.342 | 0.260 |
|  |  |  |  |  |  |
| Right precentral gyrus  Preparation of passive movement with own hand feedback | SAPS_I | 0.033 | 0.891 | 0.033 | 0.266 |
|  | SAPS_14 | 0.125 | 0.598 | 0.126 | 0.080 |
|  | SAPS_15 | -0.046 | 0.846 | -0.046 | 0.200 |
|  | SAPS_II | -0.043 | 0.857 | -0.043 | -0.045 |
|  | SAPS_III | -0.200 | 0.397 | -0.203 | -0.003 |
|  | SAPS_IV | 0.394 | 0.086 | 0.417 | 0.306 |
|  | SAPS_res | 0.281 | 0.230 | 0.289 | 0.178 |
|  | SANS_I | -0.157 | 0.508 | -0.159 | -0.155 |
|  | SANS_II | -0.137 | 0.565 | -0.138 | -0.081 |
|  | SANS_III | 0.051 | 0.832 | 0.051 | 0.141 |
|  | SANS_IV | -0.034 | 0.888 | -0.034 | -0.002 |
|  | SANS_V | 0.498 | **0.025** | 0.547 | 0.378 |
|  | SANS_total | -0.011 | 0.962 | -0.011 | 0.005 |

Note: SAPS: scale for the assessment of positive symptoms, SAPS_I: hallucinations, SAPS_II: delusions, SAPS_14: delusions of reference, SAPS_15: delusions of being controlled, SAPS_III: bizarre behavior, SAPS_IV: positive formal thought disorder, SAPS_res: residual positive symptom (SAPS_III + SAPS_IV + SAPS_V), SANS: scale for the assessment of negative symptoms. SANS_I: affective flattening or blunting, SANS_II: alogia, SANS_III: avolition/apathy, SANS_IV: anhedonia/asociality, SANS_V: attention, SANS_total (SANS_I+SANS_II+SANS_III+SANS_IV+SANS_V). Bold values represent significant correlation (p < 0.05, uncorrected). A negative correlation indicates that earlier processing (high or positive value for temporal derivative (TD)) is related to less symptoms, or other was around later processing (small or negative value for TD) is related to more symptoms.

Supplementary Table 3. Partial (by controlling the total SANS score out) correlation between neural activation timing (clusters’ eigenvariates) and symptom scores in SSD patients

| Cluster consisting mainly | symptom | Pearson correlation coeficient: r | p | Effect size (Fisher’s z) | Spearman correlation coeficient: rho^1^  correlation strength: r/rho  0.00–0.10 Negligible  0.10–0.39 Weak  0.40–0.69 Moderate  0.70–0.89 Strong  0.90–1.00 Very strong |
| --- | --- | --- | --- | --- | --- |
| Right supplementary motor area  Preparation of passive movement with own hand feedback | SAPS_I | -0.273 | 0.258 | -0.280 | 0.004 |
|  | SAPS_14 | 0.035 | 0.887 | 0.035 | 0.091 |
|  | SAPS_15 | -0.542 | **0.016** | -0.607 | 0.098 |
|  | SAPS_II | 0.248 | 0.307 | 0.253 | 0.373 |
|  | SAPS_III | 0.170 | 0.486 | 0.172 | -0.041 |
|  | SAPS_IV | -0.037 | 0.879 | -0.037 | 0.282 |
|  | SAPS_res | 0.037 | 0.882 | 0.037 | 0.152 |
| Left middle temporal gyrus  Preparation of passive movement with own hand feedback | SAPS_I | -0.287 | 0.234 | -0.295 | 0.048 |
|  | SAPS_14 | 0.189 | 0.489 | 0.191 | 0.272 |
|  | SAPS_15 | -0.416 | 0.077 | -0.443 | 0.064 |
|  | SAPS_II | 0.267 | 0.270 | 0.273 | 0.384 |
|  | SAPS_III | 0.317 | 0.186 | 0.328 | 0.230 |
|  | SAPS_IV | 0.123 | 0.616 | 0.124 | 0.132 |
|  | SAPS_res | 0.248 | 0.307 | 0.253 | 0.230 |
| Right middle temporal gyrus  Preparation of passive movement with own hand feedback | SAPS_I | -0.194 | 0.426 | -0.198 | -0.205 |
|  | SAPS_14 | 0.028 | 0.910 | 0.028 | 0.083 |
|  | SAPS_15 | -0.431 | 0.066 | -0.461 | -0.029 |
|  | SAPS_II | 0.204 | 0.402 | 0.207 | 0.254 |
|  | SAPS_III | -0.013 | 0.959 | -0.013 | -0.175 |
|  | SAPS_IV | -0.344 | 0.150 | -0.358 | -0.186 |
|  | SAPS_res | -0.326 | 0.174 | -0.338 | -0.295 |
| Right precentral gyrus  Preparation of active movement with own hand feedback | SAPS_I | -0.114 | 0.641 | -0.115 | 0.172 |
|  | SAPS_14 | -0.589 | **0.008** | -0.676 | -0.391 |
|  | SAPS_15 | -0.564 | **0.012** | -0.638 | -0.168 |
|  | SAPS_II | 0.102 | 0.678 | 0.102 | -0.260 |
|  | SAPS_III | 0.010 | 0.967 | 0.010 | 0.030 |
|  | SAPS_IV | -0.531 | **0.019** | -0.591 | -0.400 |
|  | SAPS_res | -0.490 | **0.033** | -0.537 | -0.387 |

Note: SAPS: scale for the assessment of positive symptoms, SAPS_I: hallucinations, SAPS_II: delusions, SAPS_14: delusions of reference, SAPS_15: delusions of being controlled, SAPS_III: bizarre behavior, SAPS_IV: positive formal thought disorder, SAPS_res: residual positive symptom (SAPS_III + SAPS_IV + SAPS_V), SANS: scale for the assessment of negative symptoms. SANS_I: affective flattening or blunting, SANS_II: alogia, SANS_III: avolition/apathy, SANS_IV: anhedonia/asociality, SANS_V: attention, SANS_total (SANS_I+SANS_II+SANS_III+SANS_IV+SANS_V). Bold values represent significant correlation (p < 0.05, uncorrected). A negative correlation indicates that earlier processing (high or positive value for temporal derivative (TD)) is related to less symptoms, or other was around later processing (small or negative value for TD) is related to more symptoms.

Supplementary Table 4 Correlation between neural activation durations (clusters’ eigenvariates) and symptom scores in SSD patients

| Cluster consisting mainly | symptom | Pearson correlation coeficient: r | p | Effect size (Fisher’s z) | Spearman correlation coeficient: r/rho^1^  correlation strength: r/rho  0.00–0.10 Negligible  0.10–0.39 Weak  0.40–0.69 Moderate  0.70–0.89 Strong  0.90–1.00 Very strong |
| --- | --- | --- | --- | --- | --- |
| Right supplementary motor area  Preparation of active movement with own hand feedback | SAPS_I | 0.059 | 0.805 | 0.059 | 0.016 |
|  | SAPS_14 | -0.124 | 0.601 | -0.125 | 0.195 |
|  | SAPS_15 | -0.024 | 0.919 | -0.024 | -0.119 |
|  | SAPS_II | -0.451 | **0.046** | -0.486 | -0.369 |
|  | SAPS_III | -0.275 | 0.240 | -0.283 | -0.124 |
|  | SAPS_IV | -0.066 | 0.781 | -0.066 | -0.037 |
|  | SAPS_res | -0.173 | 0.466 | -0.175 | -0.041 |
|  | SANS_I | 0.216 | 0.361 | 0.219 | 0.280 |
|  | SANS_II | -0.679 | **<0.001** | -0.828 | -0.644 |
|  | SANS_III | -0.157 | 0.508 | -0.159 | -0.127 |
|  | SANS_IV | -0.441 | 0.051 | -0.474 | -0.335 |
|  | SANS_V | 0.103 | 0.664 | 0.104 | 0.099 |
|  | SANS_total | -0.373 | 0.105 | -0.392 | -0.335 |
| Right supplementary motor area  Preparation of passive movement with own hand feedback | SAPS_I | -0.282 | 0.229 | -0.289 | -0.282 |
|  | SAPS_14 | -0.207 | 0.381 | -0.210 | -0.160 |
|  | SAPS_15 | -0.369 | 0.110 | -0.387 | -0.226 |
|  | SAPS_II | -0.524 | **0.018** | -0.582 | -0.394 |
|  | SAPS_III | 0.288 | 0.218 | 0.296 | 0.392 |
|  | SAPS_IV | -0.105 | 0.,658 | -0.106 | 0.005 |
|  | SAPS_res | 0.020 | 0.932 | 0.020 | 0.135 |
|  | SANS_I | -0.121 | 0.611 | -0.122 | -0.088 |
|  | SANS_II | -0.379 | 0.100 | -0.398 | -0.171 |
|  | SANS_III | -0.426 | 0.061 | -0.455 | -0.302 |
|  | SANS_IV | -0.477 | **0.034** | -0.519 | -0.352 |
|  | SANS_V | 0.005 | 0.985 | 0.005 | -0.020 |
|  | SANS_total | -0.516 | 0.020 | -0.571 | -0.400 |
| Left middle temporal gyrus  Preparation of active movement with own hand feedback | SAPS_I | -0.149 | 0.532 | -0.150 | -0.042 |
|  | SAPS_14 | 0.004 | 0.987 | 0.004 | -0.075 |
|  | SAPS_15 | 0.069 | 0.773 | 0.069 | -0.155 |
|  | SAPS_II | -0.279 | 0.233 | -0.287 | -0.250 |
|  | SAPS_III | -0.289 | 0.216 | -0.298 | -0.112 |
|  | SAPS_IV | 0.063 | 0.793 | 0.063 | -0.062 |
|  | SAPS_res | -0.060 | 0.800 | -0.060 | -0.053 |
|  | SANS_I | 0.191 | 0.420 | 0.193 | 0.169 |
|  | SANS_II | -0.428 | 0.060 | -0.457 | -0.378 |
|  | SANS_III | -0.061 | 0.799 | -0.069 | -0.217 |
|  | SANS_IV | -0.292 | 0.212 | -0.301 | -0.233 |
|  | SANS_V | 0.487 | **0.030** | 0.532 | 0.378 |
|  | SANS_total | -0.292 | 0.212 | -0.300 | -0.240 |
| Left middle temporal gyrus  Preparation of passive with own hand feedback | SAPS_I | 0.232 | 0.326 | 0.236 | 0.212 |
|  | SAPS_14 | -0.013 | 0.956 | -0.013 | 0.023 |
|  | SAPS_15 | 0.132 | 0.580 | 0.132 | 0.234 |
|  | SAPS_II | -0.131 | 0.581 | -0.132 | -0.076 |
|  | SAPS_III | -0.100 | 0.675 | -0.100 | -0.010 |
|  | SAPS_IV | 0.165 | 0.486 | 0.167 | 0.298 |
|  | SAPS_res | 0.111 | 0.640 | 0.112 | 0.210 |
|  | SANS_I | 0.112 | 0.638 | 0.112 | 0.151 |
|  | SANS_II | -0.136 | 0.567 | -0.137 | 0.002 |
|  | SANS_III | 0.041 | 0.865 | 0.041 | 0.079 |
|  | SANS_IV | -0.155 | 0.515 | -0.156 | -0.082 |
|  | SANS_V | 0.012 | 0.960 | 0.012 | 0.060 |
|  | SANS_total | -0.022 | 0.927 | -0.022 | -0.014 |

Note: SAPS: scale for the assessment of positive symptoms, SAPS_I: hallucinations, SAPS_II: delusions, SAPS_14: delusions of reference, SAPS_15: delusions of being controlled, SAPS_III: bizarre behavior, SAPS_IV: positive formal thought disorder, SAPS_res: residual positive symptom (SAPS_III + SAPS_IV + SAPS_V), SANS: scale for the assessment of negative symptoms. SANS_I: affective flattening or blunting, SANS_II: alogia, SANS_III: avolition/apathy, SANS_IV: anhedonia/asociality, SANS_V: attention, SANS_total (SANS_I+SANS_II+SANS_III+SANS_IV+SANS_V). Bold values represent significant correlation (p < 0.05, uncorrected).

Partial correlation analyses of duration and symptom and symptom scores by partially total negative symptom scores out have revealed no correlation.

References

1. Schober P, Boer C, Schwarte LA. Correlation Coefficients: Appropriate Use and Interpretation. *Anesth Analg*. 2018;126(5):1763-1768. doi:10.1213/ANE.0000000000002864
